# Forecasting national CO_2_ emissions worldwide

**DOI:** 10.1038/s41598-024-73060-0

**Published:** 2024-09-28

**Authors:** Lorenzo Costantini, Francesco Laio, Manuel Sebastian Mariani, Luca Ridolfi, Carla Sciarra

**Affiliations:** 1 CENTAI, Turin, Italy; 2 DIATI, Politecnico di Torino, Turin, 10129 Italy; 3 URPP Social Networks, University of Zurich, Zurich, CH-8050 Switzerland; 4 Institute of Fundamental and Frontier Sciences, University of Electronic Science and Technology of China, Chengdu, 611731 People’s Republic of China

**Keywords:** Environmental impact, Environmental economics, Environmental impact

## Abstract

Urgent climate action, especially carbon emissions reduction, is required to achieve sustainable goals. Therefore, understanding the drivers of and predicting $\hbox {CO}_2$ emissions is a compelling matter. We present two global modeling frameworks—a multivariate regression and a Random Forest Regressor (RFR)—to hindcast (until 2021) and forecast (up to 2035) $\hbox {CO}_2$ emissions across 117 countries as driven by 12 socioeconomic indicators regarding carbon emissions, economic well-being, green and complexity economics, energy use and consumption. Our results identify key driving features to explain emissions pathways, where beyond-GDP indicators rooted in the Economic Complexity field emerge. Considering current countries’ development status, divergent emission dynamics appear. According to the RFR, a −6.2% reduction is predicted for developed economies by 2035 and a +19% increase for developing ones (referring to 2020), thus stressing the need to promote green growth and sustainable development in low-capacity contexts.

## Introduction

Greenhouse gas emissions, including those of carbon dioxide, methane, and nitrogen oxides, are the leading anthropogenic causes of global warming^1,2^, one of the most compelling issues of our time according to the United Nations’ Sustainable Development Agenda^3,4^. Carbon dioxide ($\hbox {CO}_2$), in particular, is straightforwardly related to human economic activities such as energy and industrial production^2,5^, as a by-product of fossil fuels’ combustion. In accordance with the Paris Agreements^6^ signed in 2015, aiming to limit $\hbox {CO}_2$ emissions (and, thus, global warming), many countries worldwide set ambitious carbon emissions targets within the century, putting efforts towards emissions’ compensation and reduction toward a neutral carbon budget. Among the many, for example, the European Union and its Member States set a net-zero goal (i.e., balanced emissions respecting the natural carbon cycle) by 2050^7^; the same goal is set by 2060 for China^8^. Nevertheless, despite such political ambitions, $\hbox {CO}_2$ emissions are still increasing^3^.

To develop effective environmental policies and practices to reduce $\hbox {CO}_2$ emissions, reliable models estimating future carbon emissions are required^9^. In this direction, previous research works dealt with $\hbox {CO}_2$ forecasting pursuits, providing analyses focused on a single country^5,10,11^ (e.g., China^5,10^) or a limited group of countries^2,9,12–16^, such as the top-25 economies worldwide^12^. Previous works generally fit the studied forecasting approach (e.g., trend analyses^12^, multivariate regressions^14,15^, neural networks^9,10,17^, and grey models^16^) on each considered economy, providing country-specific models. These works^2,5,9–15,17^ consider predictors related with economic well-being, energy use, and urbanization to address $\hbox {CO}_2$ emissions. The choice of these predictors is supported by previous studies on the economic-environmental nexus, especially considering the Environmental Kuznets Curve (EKC)^18,19^. In the EKC approach, as countries increase their economic well-being, their $\hbox {CO}_2$ emissions rise until a turning point and then decrease, drawing an inverted U shape in the emissions-economic well-being plane. As years passed, the EKC scheme was improved by including data regarding energy use^20^, renewable energy sources^21,22^, urbanization^9^, and Research and Development (R&D)^23,24^, finding a significant correspondence with carbon emissions. However, some studies highlighted that the EKC framework is not suitable for some case studies^25,26^.

From 2017 upfront, Economic Complexity (EC) metrics feature within the EKC scheme^27–31^. Economic Complexity is a data-driven approach measuring countries’ economic sophistication from trade or production data^32–36^. Metrics derived from the EC framework detailed countries’ economic growth^37^, income inequalities^38^, and countries’ $\hbox {CO}_2$ emissions^27–31^. Moreover, Fraccascia et al.^39^ and Mealy and Teytelboym^40^ applied Economic Complexity methods to shed new light on the export of products with environmental benefits (called *green products*), which have a pivotal role in designing sustainable economic paths^41,42^. In fact, Mealy and Teytelboym^40^ showed a significant correspondence among the export of green products and low $\hbox {CO}_2$ emissions, high environmental patenting rates, and strong environmental policies. In this research panorama, forecasting models considering EC and green economy metrics to forecast $\hbox {CO}_2$ emissions are missing^27^.

Against this background, our work aims at (i) developing reliable global models to predict future countries’ $\hbox {CO}_2$ emissions and (ii) identifying crucial features leading to carbon emissions at the national scale, providing government and international institutions with indications about which features monitor for future environmental impacts. Considering the complex dynamics and modeling approaches for $\hbox {CO}_2$ budgeting^3^, we argue that an inter-country perspective on emissions and economic activities may gain insightful information for carbon-neutrality strategic planning. There are several pros to designing a global forecasting approach. Firstly, only one global model is required to estimate the carbon emissions of all countries. Secondly, the parameters of a global modeling approach are fitted considering all economies in the sample. Thus, the characteristics of all countries are considered to evaluate the final effect of each feature on the models’ output. Country-specific models do not present this property: since the model is trained only on the country at hand, the resulting coefficients do not account for other countries’ influences. Thirdly, global models can be used to retrieve a feature ranking that holds across all countries in the dataset. To strengthen the predictive capacity of the proposed global modeling frameworks, we included empirical indicators rooted in the Economic Complexity theory to test whether these indices can potentially address future countries’ environmental impacts^27^ and control for standard features such as Gross Domestic Product and renewable energy consumption.

Building on the work of Tacchella et al.^37^, we used the description of the state of an economy according to the considered features to provide estimations of national carbon emissions at different $\Delta t$ years in the future. We started by collecting data regarding $\hbox {CO}_2$ emissions, EC metrics, green products, socioeconomic indicators (such as the per-capita Gross Domestic Product), and energy-related variables (e.g., energy consumption per capita) for 117 countries worldwide from 1995 to 2020. From those, we developed two modeling frameworks (a classical multivariate regression and a Random Forest Regressor) predicting countries’ $\hbox {CO}_2$ emissions. We trained each forecasting approach considering all countries and features in our data for predicting countries’ $\hbox {CO}_2$ emissions ranging between 1 and 15 years in the future. Therefore, the resulting model is country-independent and provides $\hbox {CO}_2$ forecasting for all the economies in the analyses. Considering the models’ predictive performances, we presented an in-depth analysis of the information content of non-trivial features, such as those about the Economic Complexity field. Finally, we estimated the $\hbox {CO}_2$ emissions until 2035 for the countries in our sample. Our results show different future emissions pathways depending on countries’ economic development, unveiling non-trivial global dynamics for which we call for urgent action toward sustainable development, climate justice, and just transition.

## Results

### Global modeling approaches for $\hbox {CO}_2$ emissions forecasting

This study compares two global approaches to hindcast (in past years) and forecast (in future years) $\hbox {CO}_2$ emissions from year *t* to year $t+\Delta t$. Both frameworks aim to estimate the objective function *f* which relates the input features $x_i$ (embedded in the vector $\overrightarrow{x}$) to carbon emissions at $t+\Delta t$, i.e., ${CO_2(c,t+\Delta t)=f(\overrightarrow{x}(c,t))}$. The function *f* is estimated considering the features of all countries, providing a unique global model. $\hbox {CO}_2$ estimations for country *c* are obtained when the input vector contains the predictors for country *c*. Using as input countries’ characteristic features at the year *t*, one $\Delta t$-specific model is developed for each approach, resulting in $\hbox {CO}_2$ estimations at year $t + \Delta t$ for all the countries in our sample ($\Delta t$ ranges from 1 to 15 years with step equal to 1 year).

Among the possible models, we chose a multiplicative regression model and a Random Forest Regressor^43^ (RFR) for the following reasons. Regression models are widely used in forecasting exercises due to their ease of use and robustness, exploiting the impact of different variables over a dependent one^15^. However, they are limited in addressing complex and non-linear relationships across input features^44^, which the Random Forest Regressor allows instead. The Random Forest model is a machine-learning algorithm, whose output is the average of several decision trees (i.e., the *forest*)^43^. Alongside the chance to consider non-linear relationships, a further advantage of the RFR pertains to its optimal performances concerning possible over-fitting and features’ deletion, scaling, and collinearity issues^43,44^. Finally, both selected modeling approaches are associated with reliable criteria to rank their input features (or predictors, equivalently). Specifically, we adopted a forward step-wise feature selection algorithm^45^ for the multiplicative regression approach and a feature ranking method based on the mean decreased impurity^46,47^ for the RFR (further details are in the Data and methods section).

The multiplicative regression and RFR combine the 12 features reported in Table 1 to forecast carbon emissions. All these features contribute to addressing future countries’ emissions pathways. Consider previous values of the target variable (i.e., per-capita $\hbox {CO}_2$ emissions, $\hbox {CO}_2$) is a standard practice in forecasting studies^48^, while per-capita Gross Domestic Product (GDPpc) is linked to $\hbox {CO}_2$ emissions through the EKC^19^. Per-capita energy consumption (EnConspc) increases carbon emissions while renewable energy consumption (RenEnCons) decreases^20–22^. Urban Population (UP) increases $\hbox {CO}_2$ emissions in developing countries while decreases in developed economies^49^. We also included the Human Development Index since it was used to design future emissions scenarios at the global scale^50^. Eventually, Economic Complexity (Economic Complexity Index^33^, ECI, economic Fitness^34^, F, and Generalized Economic Complexity index^35^, GEN) and green economy (Greeness^24^, GE, and Green Complexity Index^40^, GCI) indicators proved to be determinant factors detailing (green) economic growth^35,37,40^, income inequality^38^ and carbon emissions^27–29,31^. Regarding countries’ innovation practices, we adopted the RDE^24^, a data-driven index summarizing the R&D in countries’ export basket that is highly correlated with standard innovation indicators such as the Gross Expenditure in R&D. Moreover, as the value of the RDE increases, the $\hbox {CO}_2$ intensity in the export of countries decreases^24^, supporting the concept that innovation helps achieve sustainable goals^23,51,52^. Including Economic Complexity and green economy metrics in the analysis allows us to unveil their $\hbox {CO}_2$ predictive potential. Table S1 of Supplementary Information (SI) shows a statistical description of these features between 1995 and 2020 and the Data and methods section reports further details.

We collected complete data records of the mentioned features for 117 countries between 1995 and 2020, totaling 9 low-income, 37 lower-middle-income, 29 upper-middle-income, and 42 high-income countries in the World Bank income classification. Table S2 of Supplementary Information (SI) details the countries considered in this work. To train and validate the proposed forecasting approaches, we split the dataset summarized in Table 1 into training and testing sets. The training set includes all countries and input features between 1995 and 2000, while the testing set covers input years from 2001 to 2006 (we refer the reader to Figure S1 of SI and Data and methods for further details). In other words, we formulated fifteen $\Delta t$-specific models for each modeling structure (i.e., multiplicative regression and RFR) and used the $\Delta t$ to refer to the specific forecasting model.

As a benchmark, we considered the *persistence*, i.e., we assumed that countries’ per-capita $\hbox {CO}_2$ emissions at year $t+\Delta t$ remained the same as those at year *t*. Therefore, persistence simulates the inertia of the economic and industrial systems, with associated carbon emissions^53,54^.

**Fig. 1:**
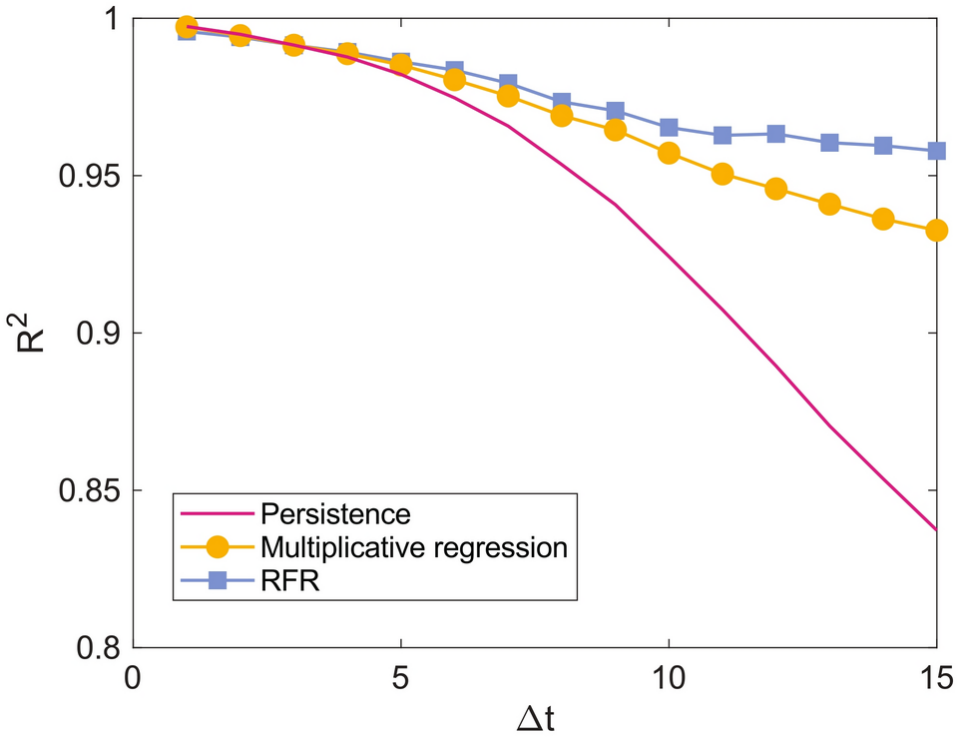
The determination coefficient ($R^2$, Equation (4)) computed between real and predicted $\hbox {CO}_2$ emissions in the testing set (i.e., input data between 2001 and 2006), for each $\Delta t$-specific model. Note that the determination coefficient is computed onto the logarithm of the emissions to balancedly weigh the residues of all countries, which span over different orders of magnitude (see Fig. 2). (To produce these results, we considered a Random Forest Regressor with 200 trees and a random state equal to 42).

**Fig. 2:**
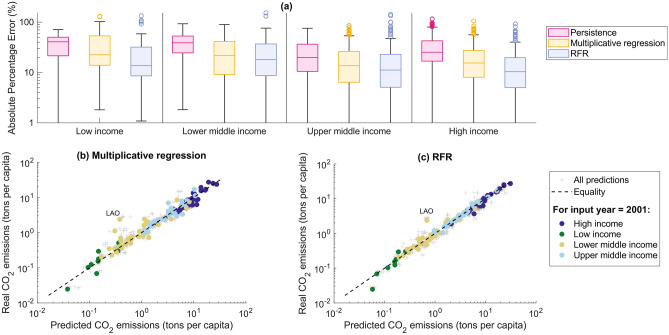
Accuracy performances for the models associated with $\Delta t=15$ years on the testing set (models’ input range between 2001 and 2006). Panel (**a**) shows the Absolute Percentage Errors (Equation (5)) for all countries (grouped by income class according to the World Bank classification) and samples in the testing set. The vertical black lines help the reader to separate the boxes according to the different income classes. For a given income class, each model-specific box shows the median, the lower and upper quartiles (box), any outliers (computed using the interquartile range, void circles), the minimum and maximum values that are not outliers (whiskers), and are color-coded according to its reference model. To improve the readability of the boxes, the y-axis was cut at 1%. Panels (**b**) and (**c**) compare actual and predicted $\hbox {CO}_2$ emissions for the multiplicative regression and RFR models, respectively. Grey pluses (+) represent all model outputs for $\Delta t=15$ years for input features in the testing set (i.e., 2001–2006). Filled dots highlight models’ predictions for input year equal to 2001 (i.e., 2016 forecasting). The color of the dots describes the income class the countries belong to. The dashed lines show the perfect match between actual and predicted values. (RFR results refer to a random state equal to 42 and 200 trees.).

**Fig. 3:**
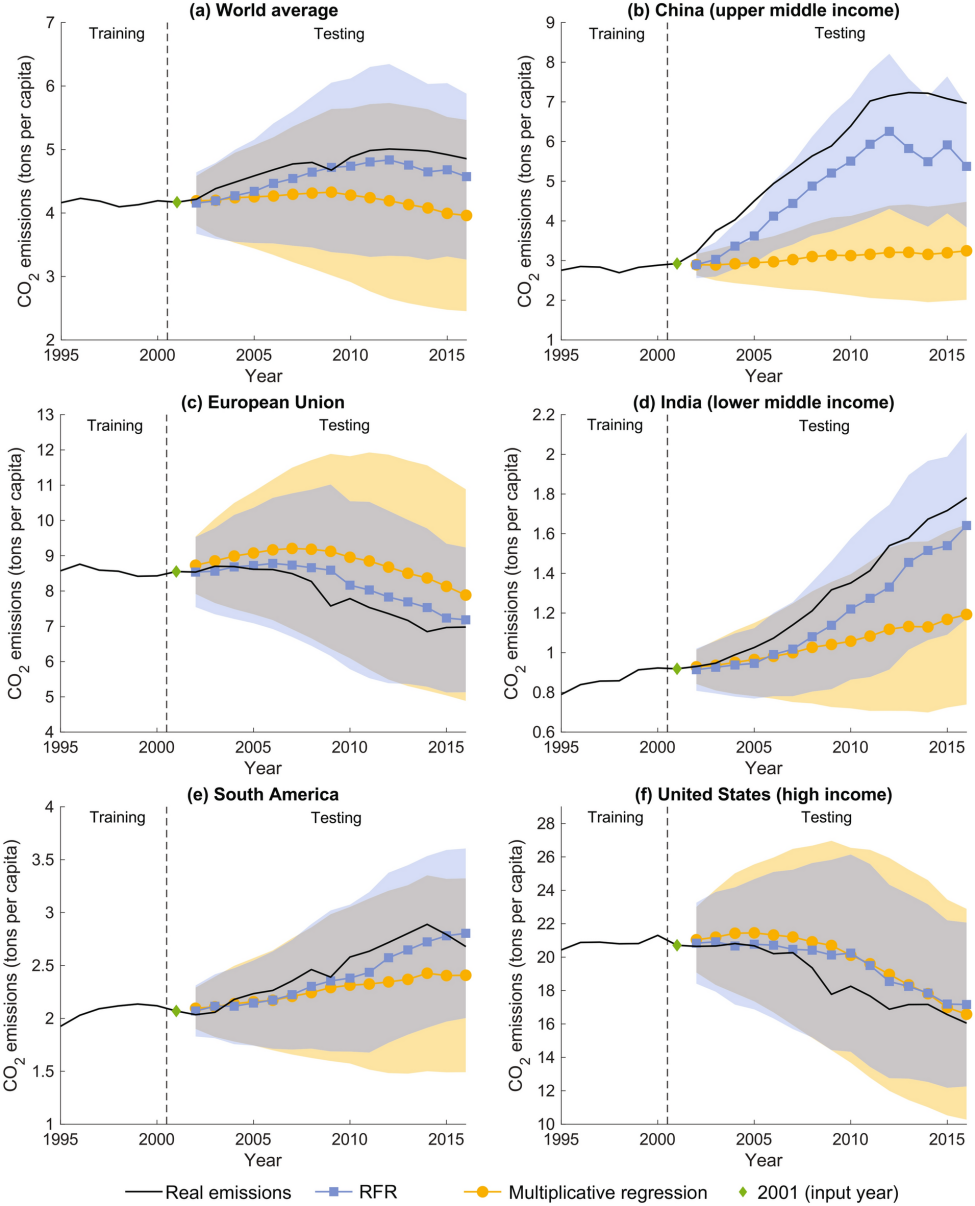
Comparison among countries’ and regions’ actual $\hbox {CO}_2$ emissions and the predicted ones by the Random Forest Regressor—RFR—(blue squares) and multiplicative regression approach (yellow circles) for world average (panel (**a**)), China (panel (**b**)), the European Union (panel (**c**)), India (panel (**d**)), South America (panel (**e**)), and the United States (panel (**f**)). Shaded regions, color-coded according to the approach they refer to, show confidence intervals associated with each prediction at 80% (overlapping confidence intervals are in grey), and the green diamond highlights the $\hbox {CO}_2$ emissions in 2001 for the country at hand (i.e., the $\hbox {CO}_2$ input data to the models). The dashed vertical lines separate the training from the testing period. (The RFR predictions are obtained considering a random state equal to 42 and 200 trees.).

**Fig. 4:**
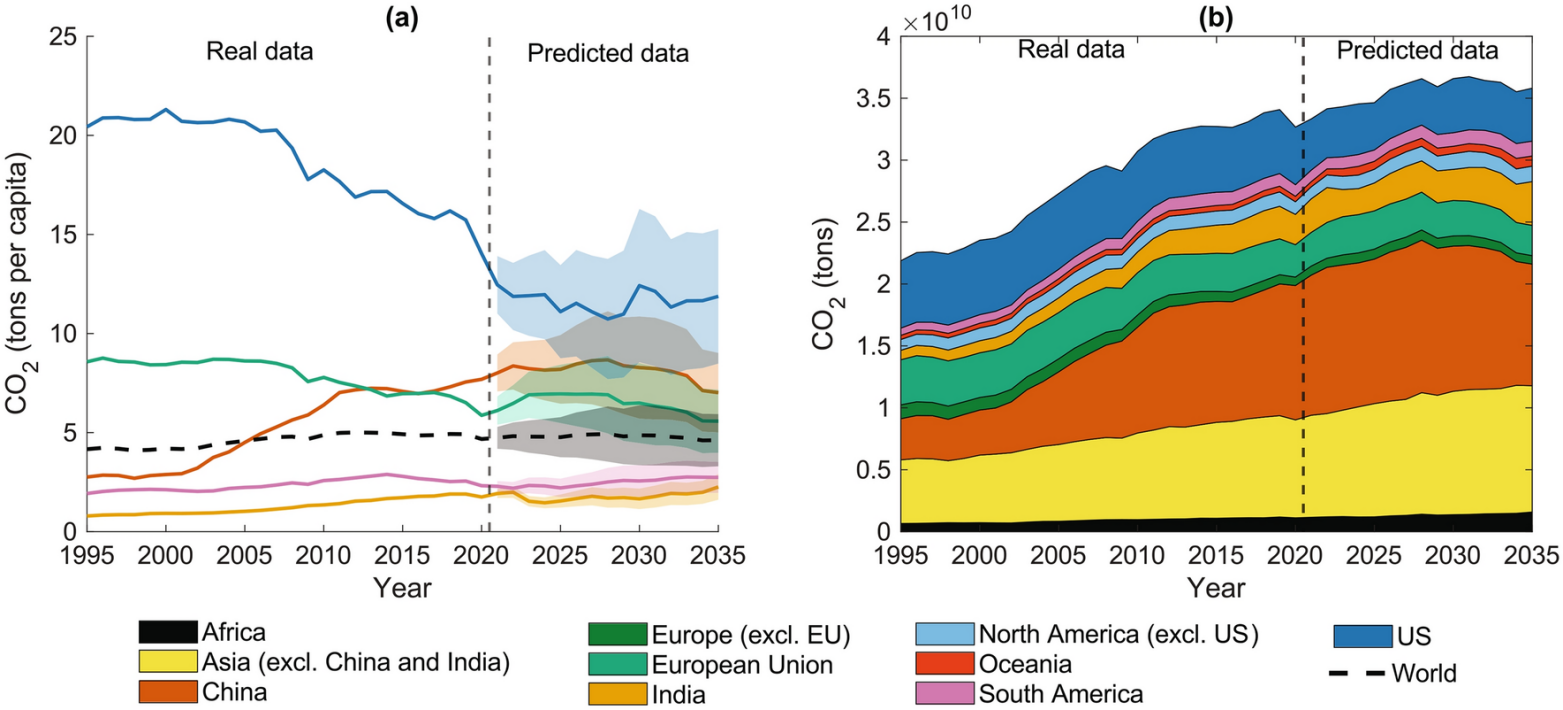
$\hbox {CO}_2$ projections until 2035 as computed by the $\Delta t$-specific models through a Random Forest Regressor modeling approach. Panel (**a**) shows the per-capita $\hbox {CO}_2$ projection for some specific countries and regions: China, the European Union, India, South America, the United States (US), and the world average (black thick dashed line). Panel (**b**) reports the $\hbox {CO}_2$ emissions at country or region scale for countries aggregated in regions as reported in Table S2 of SI. Note that panel (**b**) considers the central value of the forecasting. In both panels, dashed thin vertical lines separate the actual $\hbox {CO}_2$ emissions from the predicted ones. (The RFR predictions are obtained considering a random state equal to 42 and 200 trees.).

Aiming to show the potential of the proposed global modeling approaches, we present our results by zooming in from a general overview of models’ performances (Fig.  1) to country-specific prediction details (Figs. 2 and 3). Eventually, we study the features rankings due to the different models (Table 2) and present the 2020-2035 countries’ $\hbox {CO}_2$ projections (Fig. 4).

**Table 1:** Summary of the country-specific variables and associated data sources considered in the analyses.

Feature	Description	Source
$\hbox {CO}_2$ per-capita ($\hbox {CO}_2$)	Per-capita territorial $\hbox {CO}_2$ emissions	Global Carbon Budget^55^
Gross domestic product per-capita (GDPpc)	Gross Domestic Product divided by population at Purchasing Power Parity at constant international dollars	World Development Indicators (WDI)
Energy consumption per-capita (EnConspc)	Primary energy consumption per person in KWh	U.S. Energy Information Administration (EIA), available at https://ourworldindata.org/grapher/per-capita-energy-use
Renewable energy consumption (RenEnCons)	Share of renewable energy in total energy consumption	World Development Indicators (WDI)
Urban population (UP)	Share of people living in metropolitan areas	World Development Indicators (WDI)
Human development index (HDI)	Aggregated measure of human development, as the geometric mean of life expectancy, years in school, and gross national income per capita	United Nations, available at https://hdr.undp.org/data-center/human-development-index#/indicies/HDI
Economic complexity index (ECI)	Measure of countries’ economic complexity as the average sophistication of the products it exports^32,33^	Authors’ computation using the BACI-CEPII dataset^56^ according to Hidalgo et al.^32,33^
Economic fitness (F)	Sum of the sophistication of the products exported by a country^34^	Authors’ computation using the BACI-CEPII dataset^56^ following Tacchella et al.^34^
GENEPY index (GEN)	Measure of countries’ economic complexity coupling diversification and the similarity within export baskets^35,57^	Authors’ computation using the BACI-CEPII dataset^56^ in line with Sciarra et al.^35^
Greenness (GE)	Share of green products within the export baskets of countries^24^	Authors’ computation using the BACI-CEPII dataset^56^ and the green products data from Mealy and Teytelboym^40^ as described in Costantini et al.^24^
Green complexity Index (GCI)	Sum of the complexity of the green products exported by a country^40^	Authors’ computation using the BACI-CEPII dataset^56^, relying on the work by Mealy and Teytelboym^40^
R&D intensity embedded in countries export baskets (RDE)	Measure of the R&D embedded in countries export basket^24^	Authors’ computation using the BACI-CEPII dataset^56^ following Costantini et al.^24^

Horizontal lines separate the blocks of features as described in the Data and methods section.

### Models’ aggregate performances

Our first result compares the multiplicative regression and Random Forest Regressor with the persistence (i.e., the benchmark model that only reproduces countries’ past $\hbox {CO}_2$ emissions), showing that the proposed predicting approaches achieve higher performances. To illustrate this point, Fig. 1 shows the determination coefficient—computed on the testing set, Equation (4) in the Data and methods section—for each $\Delta t$-specific model. At a glance, we notice that the persistence shows high values of $R^2$ due to the high auto-correlation of countries’ $\hbox {CO}_2$ emissions (see Figure S2). However, including other features to address $\hbox {CO}_2$ emissions improves notably the predictions. Specifically, as Fig. 1 shows, the RFR approach presents the highest $R^2$ values, followed by the multiplicative regressions. Moreover, these forecasting frameworks decrease their $R^2$ values slower than the persistence as the $\Delta t$ increases. This result points to the essential role of nonlinear dependencies among the considered features to unveil future national $\hbox {CO}_2$ emissions.

### Models’ performances for different income classes

We deepen the analysis of the prediction accuracy by grouping countries according to the World Bank income classification. The income classification helps the reader to contextualize models’ performances while framing countries according to the most common indicator of economic well-being (i.e., income). Figure 2a shows the country-specific Absolute Percentage Errors (APE, Equation (5)) for the models associated with $\Delta t=15$ years in the testing set. As expected, the RFR generally presents the lowest APE values among the proposed predictive approaches for countries in all income classes, followed by the multiplicative regression. The latter model presents APE values similar to those due to the RFR, especially for countries with lower-middle and upper-middle-income. Conversely, the persistence shows the highest relative error for countries in all income classes, confirming that the proposed modeling approaches outperform the benchmark model (i.e., the persistence) in terms of prediction accuracy. Thus, our models detect a non-constant dynamic of $\hbox {CO}_2$ emissions worldwide that improves models’ forecasting performances.

Figures 2b and c compare countries’ actual and predicted $\hbox {CO}_2$ emissions for the multiplicative regression and RFR models, respectively. Among the $\hbox {CO}_2$ forecasting by the models for $\Delta t=15$ years, we highlight the predictions with input year $t=2001$ (i.e., the first year of the testing set that is a representative example of the models’ behavior), thus predicting the country-specific values of $\hbox {CO}_2$ emissions for 2016. These scatter plots help better frame the information embedded in the Absolute Percentage Error (Fig.  2a). As expected, the multiplicative regression and RFR models (Fig. 2b and c, respectively) do not present systematic over- or under-estimates of countries’ $\hbox {CO}_2$ emissions despite having designed global modeling frameworks rather than country-specific ones. The RFR provides the most accurate predictions (points are generally closer to the bisectrix than the multiplicative regression). Some exceptions exist due to country-specific peculiarities. An example is Laos (LAO): both models under-estimate Laos’ future $\hbox {CO}_2$ emissions, which can be reasoned by studying Laos’ $\hbox {CO}_2$ time series compared to those of the other countries. Laos has the highest increment in relative emissions among all considered economies (refer to Figure S3 of SI). This specific behavior is almost unique within the countries in our sample. For this reason, the developed models fail to detect this behavior, leading to high forecasting errors.

In brief, the RFR model for $\Delta t = 15$ years outperforms the persistence (and multiplicative regression model) in terms of prediction accuracy.

### Models’ performances in reproducing countries’ and regions’ time series

We show the potential of the proposed modeling approaches in forecasting $\hbox {CO}_2$ emissions at the regional and local scale by comparing actual and predicted per-capita $\hbox {CO}_2$ emissions time series. To this aim, we considered the world average of our sample, highlighting some countries and world regions representative of different income and geographic locations: China, the European Union, India, South America, and the United States (US). Figure 3 shows the actual and predicted $\hbox {CO}_2$ time series for the multiplicative regression and RFR, considering predictions with input year equal to 2001, over the entire $\Delta t$ range (thus, up to 2016). The $\hbox {CO}_2$ emissions for the world average (Fig. 3a), European Union (Fig. 3c), and South America (Fig. 3e) were computed as the average weighted by populations of countries belonging to the region at hand (Table S2 of SI). Population data were retrieved from the World Development Indicators. We include possible emission pathways by computing the 80% confidence interval around the predicted values, considering their distribution to be normal (refer to the SI for further details).

The world $\hbox {CO}_2$ emissions average (Fig. 3a) is within the confidence bands at 80% for both the developed approaches. However, the RFR method better follows the actual time series than the multiplicative regression. Specifically, the RFR outperforms the multiplicative regression because it provides better forecasting of China’s and India’s $\hbox {CO}_2$ emissions (Fig. 3b and d), which have about 40% of the total population. As Fig. 3b and d show, China and India notably increased their $\hbox {CO}_2$ emissions, determining the low performances of the multiplicative regression approach when sharp changes in $\hbox {CO}_2$ emissions occurred. For developed economies, such as countries in the European Union (Fig. 3c) and the United States (Fig. 3f), actual and predicted time series agree for both approaches. More in-depth, the proposed frameworks catch the European Union’s decreasing trend of per-capita $\hbox {CO}_2$ emissions, but RFR predictions are slightly better. Eventually, the RFR presents more accurate $\hbox {CO}_2$ emissions forecasting also for countries in South America, despite the actual emissions being within the confidence bands at 80% for both the proposed modeling approaches.

In a nutshell, the multiplicative regression and RFR provide good estimates of future $\hbox {CO}_2$ emissions. However, the RFR achieves more accurate forecasting for countries belonging to any income class and for all $\Delta t$s.

### Ranking countries’ features

Models’ ability in the prediction task can be reasoned in their use of the features. We adopted different feature ranking approaches depending on the predictive method at hand. A forward step-wise feature selection algorithm works well for the multiplicative regressive model^45^, while the mean decreased impurity works for the RFR (refer to^43,46,47^ for further details). Table 2 reports the features sorted according to the average ranking among the developed forecasting approaches (i.e., multiplicative regression and RFR), associated with $\Delta t$s ranging from 1 to 15 years. The most relevant predictor to forecast $\hbox {CO}_2$ emissions at time $t+\Delta t$ is the $\hbox {CO}_2$ emissions at time *t* for all predictive methods considered in this work, coherently with the high correlation observed among countries’ $\hbox {CO}_2$ emissions at different times (see Figure S2 in SI). For both the developed predictive frameworks, Economic Complexity metrics are among the top 4 features. Specifically, Fitness (F) is the second and fourth most important feature for the multiplicative regression and RFR, respectively. We recall that the Fitness measures countries’ economic performances by coupling the number of exported products with their sophistication^34^, and this Economic Complexity metric proved to be able to forecast countries’ economic growth in terms of GDP^37^. This result shows that economic Fitness helps forecast also countries’ carbon emissions. Renewable energy consumption (RenEnCons) is a significant predictor for the developed approaches, in line with previous research works^21,22^. In fact, it is in position 5 and 3 for the multiplicative regression and RFR, respectively. Countries’ per-capita GDP is in the 6th and 5th ranking position for the multiplicative regression and RFR, respectively.

Despite the previously mentioned similarity among the top-half features rankings, there are some differences. Focusing on the differences among the Economic Complexity metrics, the ECI is at the third position of the ranking for the multiplicative regression. We remind that ECI groups countries according to the sophistication of the products they export (limiting this clustering to two subsets of products’ sophistication)^35,58^ and that it is the most used Economic Complexity indicator to address the economic system-$\hbox {CO}_2$ nexus^27–29,31^. For the RFR model, instead, we find the Generalized Economic Complexity (GENEPY, shortened as GEN in this work) index in the second position of the ranking. The GEN ranks countries coupling countries’ diversification and the similarities among their export baskets, providing a framework to detail countries’ (economic) development trajectories^35^ and Sustainable Development Goals achievements^59^. Eventually, the multiplicative regression considers the shares of green products (greenness, GE) as valuable information to unveil future $\hbox {CO}_2$ emissions. Greenness is at the fourth position of the ranking and, in line with a previous study, the export of green products correlates with low $\hbox {CO}_2$ emissions^40^. Conversely, for the RFR, the R&D embedded in countries’ export basket (RDE) is at the 6th ranking position. Generally, as the RDE increases, countries’ greenness increases, and $\hbox {CO}_2$ emissions per exported dollar decreases^24^. Therefore, this result enforces previous works sustaining that investments in R&D help promote sustainable development practices^24,52,60^.

**Table 2:** Mean variables’ ranking for each forecasting model among all the considered $\Delta t$.

Ranking	Multiplicative regression	RFR
1	$\hbox {CO}_2$	$\hbox {CO}_2$
2	F	GEN
3	ECI	RenEnCons
4	GE	F
5	RenEnCons	GDPpc
6	GDPpc	RDE
7	GEN	GE
8	RDE	ECI
9	GCI	EnConspc
10	HDI	HDI
11	UP	GCI
12	EnConspc	UP

Variables’ order is computed according to a forward step-wise feature selection algorithm for the multiplicative regression and with the mean decrease impurity for the Random Forest Regressor (RFR).

Against the general overview of features’ ranking presented in Table 2, we discuss how the specific models for $\Delta t=15$ years (i.e., those studied in Fig. 2) combine the considered predictors.

Table S3 of SI reports the coefficients of the multiplicative regression model. We limit our comments to some examples of statistically significant predictors. Past carbon emissions are the most relevant feature and present a positive coefficient. The ECI presents a negative coefficient; thus, as countries orient their export basket toward sophisticated goods, they tend to reduce carbon emissions. Finally, renewable energy consumption has a negative coefficient which might be explained by the role of investments in renewable energies for reducing $\hbox {CO}_2$ emissions^21,22^.

For the RFR model, we used the SHapley Additive exPlanations (SHAP)^61^ to interpret the outputs of the RFR model for $\Delta t=15$ years. Specifically, the SHAP framework assigns each prediction an importance value for each feature, called SHAP value^61^. In this way, one can detail the model output by summing the SHAP values for each feature and the average prediction of the model at hand^61^. Figure S4 of SI shows the SHAP values of the RFR model for the considered features. As for the multiplicative regression, past $\hbox {CO}_2$ emissions significantly affect the predictions. Economic Complexity indicators are at the second and third position of the ranking, where the Generalized Economic Complexity index and Fitness—respectively—are observed. In general, as countries increase their GEN and Fitness values, the associated SHAP values tend to decrease, suggesting a correlation with $\hbox {CO}_2$ emissions. This observation paves the way for further analysis linking the EC metrics to $\hbox {CO}_2$ emissions.

To sum up, in both cases, non-trivial variables from the Economic Complexity field, green products, and R&D play a key role in improving the models’ predictions.

### Predicting $\hbox {CO}_2$ emissions until 2035

In light of the valuable outcomes of the models in detailing $\hbox {CO}_2$ emissions during the years considered in the testing set, we use the RFR and multiplicative regression approaches to forecast countries’ emissions from 2020 to 2035. Specifically, we trained the models between 1995 and 2000; then, we gave them the year 2020 as input and evaluate the outcomes. Here, we comment on RFR’s projection (Fig. 4), i.e., the approach presenting better predictive performances on the testing set than the multiplicative regression. Similar results hold for the multiplicative regression, as Figure S5 of SI shows. Confidence intervals were computed considering the $\Delta t$-specific coefficient of variation calculated on the testing set (refer to the Supplementary Information for further details).

Figure 4a shows the per-capita $\hbox {CO}_2$ forecasting for the six world regions reported in Fig. 3. Note that—in this case—the world and region average values were computed as the weighted average of the countries’ $\hbox {CO}_2$ predictions by the RFR model and the median population projection proposed by the United Nations (freely available at https://population.un.org/wpp/). In Fig. 4a, the reader can note that the predicted world average (black dashed thick line) might not change significantly from 2020 to 2035. However, our model detects different future behaviors at the country and region scale. For example, according to the RFR modeling approach, developed economies of the European Union might show a decreasing trend in $\hbox {CO}_2$ emissions while remaining above the world average. Instead, predictions for India and countries in South America might show below-average $\hbox {CO}_2$ emissions and increasing per-capita emissions by 2035. The models’ predictions for the United States suggest a static evolution of $\hbox {CO}_2$ emissions, where per-capita $\hbox {CO}_2$ emissions in 2035 might decrease by 15% compared to 2020. Finally, China’s predicted emissions are in line with an Environmental Kuznets Curve: they might increase up to a peak around 2028, and present a decreasing trend afterward. This peculiar dynamics has been further explored using the SHAP values (refer to figure S6 of SI)^61^. As expected, $\hbox {CO}_2$ emissions are the most relevant feature and set the final emission forecasting to the right order of magnitude concerning the average model’s predictions. Fitness and GEN are the second and third most important features for China’s predictions, especially from 2028 onward. The contribution of these features to China’s forecasting is always negative, leading to a decrement in the final $\hbox {CO}_2$ emissions estimation. According to the Economic Complexity theory, the economic Fitness and Generalized Economic Complexity index capture the set of capabilities (i.e., knowledge and know-how) a country has developed^34,35,62^. Therefore, the RFR model suggests that China might have the capabilities required to reduce its $\hbox {CO}_2$ emissions starting from the end of this decade.

Figure 4b reports the projected $\hbox {CO}_2$ emissions at the country scale (i.e., countries’ per-capita $\hbox {CO}_2$ emissions were multiplied by the corresponding population estimates), aggregated in the following regions: Africa, Asia (excluding China and India), China, Europe (excluding European Union), the European Union, India, North America (excluding the US), Oceania, South America, and the United States (see Table S2 of SI for the correspondence between countries and regions). Predictions of total $\hbox {CO}_2$ emissions are in line with per-capita forecasting. For example, our predictions show that countries in the European Union (India) might slightly decrease (increase) their $\hbox {CO}_2$ emissions by 2035. According to the RFR forecasting, China’s $\hbox {CO}_2$ emissions might increment until around 2028 and decrease afterward. Although RFR predictions for the US present a decrease in the per-capita $\hbox {CO}_2$ emissions between 2020 and 2035, the projections for their national emissions are almost constant in the same period (only a -8% variation). The mismatch between the US’s national and per capita $\hbox {CO}_2$ projections is related to the population growth expected from 2020 to 2035 (see  https://population.un.org/wpp/). We comment on global $\hbox {CO}_2$ emissions cumulating the RFR’s predictions for the single countries. RFR’s $\hbox {CO}_2$ emissions estimations might reach a maximum around 2028 (the same year as China, i.e., the largest $\hbox {CO}_2$ emitter), followed by a moderate decrement. Within this general framework, the RFR’s forecasting presents that Asian countries might continue increasing carbon emissions, except China.

## Discussion and conclusions

Aiming to provide novel global modeling approaches to $\hbox {CO}_2$ forecasting, our results show that (i) the multiplicative regression and Random Forest approaches have better predictive performances in $\hbox {CO}_2$ forecasting (as shown in Figs. 1 and 2) than the persistence; (ii) the Random Forest Regressor presents higher accuracy values than a classical multiplicative regression; (iii) these modeling frameworks rank non-trivial features from the Economic Complexity theory as crucial factors to address future countries’ $\hbox {CO}_2$ emissions; (iv) they forecast a global emission peak around 2030, followed by a slight decrement; and (v) according to models’ predictions, Asian economies might dominate the $\hbox {CO}_2$ emissions panorama in the next future.

Previous works^2,5,9–15,17,63^ generally focused on country-specific models, fitting the model’s parameters to the considered country. Our approach is different and entails developing a unique model capable of forecasting (for a specific $\Delta t$) the $\hbox {CO}_2$ emissions of all countries in our database. The advantage of the modeling frameworks we propose is to obtain a general (i.e., country-independent) ranking of the features considered throughout this work. We argue that developing global rather than country-specific models allows one to obtain more robust results, especially considering the limited data availability for the features we considered. In fact, developing such models entails finding a proper balance in the spatial and temporal coverage of the input datasets, preserving a proper number of country-specific data over time. We checked the temporal extension of the dataset herein used, finding a backward limitation to 1995, exceptionally due to the BACI-CEPII dataset^56^ from which the EC and green economy metrics were processed. This dataset covers international trade in extreme detail, reconciling information on the exporter and importer side^56^. A way to improve the temporal extension on these particular data inputs would be to data-mining the official United Nations COMTRADE dataset. Yet, the advantage would only be a 5-year enlargement of our time series, with possible losses in country coverage, since other metrics, such as the Human Development Index and Renewable energy consumption, are limited to 1990. A proper balance between the operational cost and the information gained has thus guided our choice of datasets. Despite the partially limited temporal extent, our models present a mean absolute percentage error below 25% (see Figure S7) that still entails reasonable and good forecasting performances^64^.

While models’ quality performances in hindcast can be grasped by the comparison with the observed values, at both national and global scales, other considerations shall be applied when evaluating the model’s performances in the future. We thus compare the RFR estimations (the most promising global model we propose) in the time horizon 2020-2035 with other $\hbox {CO}_2$ projections by country-specific models available in the literature^2,12,15^ (refer to Table S4 of SI for further details). In general, our estimations are consistent with earlier studies^12,15^. For example, our 2030 estimations agree with those of Köne and Büne^12^ for Brazil (0.481 Gtons vs 0.479 Gtons) and Mexico (0.612 Gtons vs 0.614 Gtons). We detected good alignment even pushing the comparison to 2035 for the same countries. According to Karakurt and Aydin^15^, Brazil’s (Mexico’s) $\hbox {CO}_2$ emissions are expected to reach 0.578 (0.598) Gtons in line with our projections (0.535 Gtons for Brazil and 0.60 Gtons for Mexico). The different methodological frameworks and input features lead to limited variations in the estimations between 2030 and 2035 that can be reasoned considering the sophistication of the economies, i.e., the Economic Complexity metrics (we remind that the Fitness, F, and Generalized Economic Complexity index, GEN, are relevant predictors for the RFR). China is a good example: our 2030 estimations are 11.7 Gtons and 9.80 Gtons in 2035. Instead, China’s projection by Karakurt and Aydin^15^ are 14.3 Gtons in 2030 and 15.6 Gtons in 2035. We argue that the high fitness and GEN values of China in 2020 (10 and 68 against a global average of 1.44 and 15.40, respectively) decrease the final output of the RFR model (especially for 2035 estimations), determining the trend inversion and explaining these differences (see Figure S4 and S6 of SI). Considering the results of the multiplicative regression model, the reasons for the different estimates are the renewable energy consumption, innovation practices, and economic sophistication, in line with previous studies^21–23,51,65,66^.

One limitation of the present work relates to unpredictable events that can influence countries’ $\hbox {CO}_2$ emissions. Our predicting approaches fit past dynamics considering $\hbox {CO}_2$ emissions, EC metrics, and socioeconomic indicators. However, national emissions may be affected by future black swans that are not in the current training dataset (e.g., a new pandemic or unprecedented green policies in some countries), which these kinds of models may fail to account for. Therefore, the current work provides $\hbox {CO}_2$ forecasting worldwide, assuming that countries’ attitudes to environmental issues and socioeconomic activities will not substantially change in the future and that emissions variations will mainly derive from the current governance structure. Nevertheless, the developed models offer the flexibility to be retrained over time and be enhanced with additional features, which may improve predictability should major structural factors change over the coming years.

Similar results hold for consumption $\hbox {CO}_2$ emissions^67^ (i.e., allocating $\hbox {CO}_2$ emissions considering international trade), as the reader can see in Figures S8–S11 and Table S5 of SI. However, comparing consumption and territorial analyses, differences in feature ranking emerge. Specifically, our analyses on territorial emissions highlight the key role of Economic Complexity metrics that measure countries’ economic specialization in certain productive sectors^33–35^. Conversely, energy-related predictors (especially energy consumption per capita) are among the top features affecting the prediction of consumption $\hbox {CO}_2$ emissions (refer to Table S5 of SI). These outcomes support previous findings stating that developed economies shift high carbon intensity activities abroad and benefit from them through international trade^67–71^. In fact, energy production and consumption practices are strongly related to fossil fuels^2,5^, which account for a notable share of total carbon emissions embedded in international trade^71^.

Our results pave the way for future research efforts addressing the economic activity-$\hbox {CO}_2$ nexus. Throughout this work, we noted a correlation between Economic Complexity metrics and future territorial $\hbox {CO}_2$ emissions. It is beyond the scope of the present work to investigate whether a causal link exists between the EC metrics and carbon emissions, and we address future research activities to this aim. Another research direction entails developing a forecasting framework addressing future countries’ territorial and consumption $\hbox {CO}_2$ emissions in sync. Such a model will shed new light on sustainable production and consumption practices. Eventually, the $\hbox {CO}_2$ estimations we presented between 2020 and 2035 provide a data-driven benchmark for future study or as the input of scenario analyses aimed at developing environmental policies at the national and international scale.

To conclude, this work provides three main contributions to the literature on $\hbox {CO}_2$ forecasting. Firstly, we developed global models (a multiplicative regression and a Random Forest Regressor) to forecast countries’ $\hbox {CO}_2$ emissions, i.e., a unique model predicts $\hbox {CO}_2$ emissions for all countries in our database. Our results highlight the satisfying predicting performances of the developed modeling frameworks, especially those of the Random Forest Regressor. Secondly, several non-trivial predictors related to the Economic Complexity framework are notable countries’ features to address $\hbox {CO}_2$ emissions. For example, the Generalized Economic Complexity index and economic Fitness are crucial features of the Random Forest Regressor: as economies become more sophisticated, they tend to reduce their carbon emissions. Thirdly, we used the proposed approaches to predict countries’ $\hbox {CO}_2$ emissions until 2035. According to the RFR forecasting, developed economies might decrease—on average – their national $\hbox {CO}_2$ emissions by 6.2% compared with 2020 emissions, while developing economies might increment $\hbox {CO}_2$ emissions by around 19%. Within this general scheme, RFR predictions for China’s national emissions might rise by around 13.5%—compared to those of 2020—until around 2028 and then might decrease by 20%—referring to 2028 emissions—between 2028 and 2035, coherently with the Environmental Kuznets Curve. The RFR model detects this emissions reduction thanks to Economic Complexity measures of economic sophistication. Therefore, our results enforce and extend the literature concerning the nexus between countries’ economic sophistication and $\hbox {CO}_2$ emissions^19,24,27–29,31^.

## Data and methods

### Data

$\hbox {CO}_2$**emissions data** For $\hbox {CO}_2$ emissions, we considered countries’ per-capita $\hbox {CO}_2$ emissions due to the combustion of fossil fuels (coal, gas, and oil), gas flaring, and cement production, retrieved from the Global Carbon Budget^55^. $\hbox {CO}_2$ emissions are accounted as territorial or consumption emissions. Territorial emissions are those emissions that occur within countries’ borders. Instead, consumption emissions include international trade in allocating $\hbox {CO}_2$ emissions. Therefore, consumption $\hbox {CO}_2$ emissions of a country are equal to its territorial emissions plus the emissions embedded in its imports and minus those due to the exports^55,67^. In the main text, we focus on territorial $\hbox {CO}_2$ emissions, while the Supplementary Information reports analyses on consumption carbon emissions. Note that countries’ $\hbox {CO}_2$ emissions at time *t* are one of the features the developed models take as input, while $\hbox {CO}_2$ emissions at time $t+\Delta t$ are the models’ output.

**Socioeconomic indicators** As for socioeconomic indicators, we considered 5 features, which we detail in Table 1 with associated data sources. These features include the Gross Domestic Product per capita (GDPpc), Human Development Index (HDI), per-capita energy consumption (EnConspc), renewable energy consumption (RenEnCons), and Urban Population (UP). The choice for these specific predictors stems from previous work supporting the linkages among these features and $\hbox {CO}_2$ emissions. GDPpc is considered a standard indicator to unveil the economy-$\hbox {CO}_2$ nexus since the introduction of the Environmental Kuznets Curve^18,19^. Since HDI provides a more general overview of countries’ socioeconomic characteristics (i.e., combining income, life expectancy, and instruction), this index was used to explain countries’ $\hbox {CO}_2$ emissions^50^. For what regards energy-related variables (i.e., EnConspc and RenEnCons), previous works showed that energy consumption is positively correlated with $\hbox {CO}_2$ emissions, negatively instead with renewable energy consumption^21,22,72^. Finally, the urban population (UP) was considered in this work in light of the high carbon emissions due to people living in cities, thus proxying economic activities^49,73,74^.

**Economic Complexity, R&D, and green economy metrics** Economic Complexity (EC) aims to measure countries’ economic development, studying the composition of their export baskets, encoded in the country-products network^33–36^. Several EC metrics were developed to accomplish this task. The best-known indices are the Economic Complexity Index (ECI)^33^, the economic Fitness (F)^34^, and the Generalized Economic Complexity index (GENEPY, shortened as GEN in this work)^35^. In brief, the ECI defines the economic sophistication of a country as the average complexity of the exported goods^33^. The Fitness combines the number of products a country exports and the products’ complexity. The GEN, instead, ranks countries’ economic performances depending on a multidimensional complexity computed through diversification (i.e., number of exported products) and similarities in the export baskets^35,57^. Moreover, studying the country-products network considering the Research and Development (R&D) intensity embedded in the traded goods, Costantini et al.^24^ developed the RDE, a novel index measuring the R&D content in countries’ export basket. All EC metrics were involved in sustainability studies about Sustainable Development Goals^59^ and $\hbox {CO}_2$ emissions^24,27–31^, finding a significant correspondence.

With the term *green economy*, we mean the trade of those products with environmental performances, also called *green products*^39,40^. Green products are those commodities reducing, limiting, and preventing environmental degradation of any kind^75^. Recalling the linkages between the export of green products and virtuous environmental performances^40^, we included green-product-related metrics in our analyses. Specifically, we used the greenness (GE) and Green Complexity Index (GCI). The greenness is the share of green products within countries’ export baskets^24^, and the Green Complexity Index describes countries’ performances in the green economy by summing the complexity of the exported green products^40^.

**Population data** Population data were retrieved from the World Development Indicators between 1995 and 2022 and from the United Nations projections (freely available at https://population.un.org/wpp/) from 2023 upfront. Countries’ population at a given time was used as a weight to compute the average $\hbox {CO}_2$ emissions of a region.

### Multiplicative regression

The multiplicative regression combines the features in a multiplicative way, described as follows:

1 $$\begin{aligned} CO_2(c,t+\Delta t)=k\prod _i x_i^{a_i}(c,t), \end{aligned}$$

where *k* is a constant, $x_i(c,t)$ represents the *i*-th feature presented in Table 1 (for country *c* at time *t*), and $a_i$ is the associated exponent. $\Delta t$ represents the prediction interval (ranging from 1 to 15 years) and a specific model is formulated for each $\Delta t$. Equation (1) can be written in an additive form considering the logarithm (base 10) of the variables:

2 $$\begin{aligned} \log CO_2(c,t+\Delta t)=\sum _i a_i\log x_i(c,t) + \log k. \end{aligned}$$

to train the multiplicative regression (see Equations (1) and (2)), all predictors that can have zeros or negative values—e.g., the Economic Complexity Index (ECI), Green Complexity Index (GCI), and renewable energy consumption (RenEnCons)—were scaled to take only positive values as follows:

3 $$\begin{aligned} x(c,t)'=x(c,t)+|\min (x)| +1, \end{aligned}$$

where $\min (x)$ indicates the minimum value for the variable at hand for any country at any time.

The multiplicative regression approach has two desirable properties. Firstly, its interpretation is simple to understand. Secondly, several criteria exist to rank variables according to their contribution. In this work, we sorted the features according to a forward step-wise feature selection algorithm^45^. In brief, we added the features to the model one at a time depending on which one determines the highest model’s determination coefficient (defined in the Evaluation of models’ performances section)^45^.

### Random Forest Regressor

The other predictive model is a Random Forest Regressor (RFR)^43^, i.e., a machine learning approach combining multiple decision trees to improve predictions’ accuracy. The final output of the RFR is the average output of the trees composing the model. Each three analyzes a bootstrap sample of the training set using a random subsample of the input features to fit the actual value of the target variable. In such a way, the generated trees are independent of each other^43^. We chose a machine learning algorithm to highlight the role of non-linear links among the considered features and $\hbox {CO}_2$ emissions. We considered a Random Forest Regressor because (i) the random forest is a method that can easily handle several variables without the need to re-scaling the inputs, (ii) it is robust to overfitting problems, and (iii) the method provides criteria to rank the features according to the mean decreased impurity. In a nutshell, the mean decreased impurity criteria measures the ability of each feature to split the input data across trees’ nodes, aiming to obtain homogeneous subsets according to the target variable. The more a feature can split the data, the higher its ranking is (refer to^46,47^ for further details). This feature-ranking approach is suitable for the data we collected because the variables are not highly correlated^46,47^ (see Figure S12 in SI). Eventually, the use of RFR (as well as other machine learning algorithms) in economic forecasting is common practice^76^. From sensitivity analyses on $\Delta t$s (i.e., for different forecasting models), and territorial and consumption $\hbox {CO}_2$ emissions, we set the number of trees in the RFR equal to 200.

### Training and testing sets

The dataset we collected (Table 1) was split into training and testing sets. Details follow, and Figure S1 accompanies the description to better understand the sets definition.

We trained all predictive models considering the whole set of countries and features presented in Table 1 between 1995 and 2000. Specifically, the models receive as input the state of all economies as described by the 12 features in Table 1 at time *t* (with *t* between 1995 and 2000 included), aiming to fit the countries’ $\hbox {CO}_2$ emissions at time $t+\Delta t$ ($\Delta t$ ranges from 1 to 15 years in the future). Therefore, fifteen $\Delta t$-specific models are formulated for each of the two chosen modeling structures (i.e., multiplicative regression and RFR). We use the $\Delta t$ to refer to the specific forecasting model.

Once we had trained the models for all $\Delta t$s, we used countries’ features between 2001 and 2006 (included) to forecast $\hbox {CO}_2$ emissions at time $t+\Delta t$, with $2001\le t \le 2006$ and $1\le \Delta t \le 15$ years. Then, we compared the output of the models with the actual $\hbox {CO}_2$ emissions to study the models’ performances. We validated the developed models by comparing actual and predicted values until 2021.

### Evaluation of models’ performances

We used the determination coefficient ($R^2$) and the Absolute Percentage Error (APE) to evaluate the models’ predictions. The determination coefficient for the model identified by a specific $\Delta t$ is

4 $$\begin{aligned} R^2(\Delta t)=1-\frac{\sum _{c,t} (CO_2^{pred}(c,t+\Delta t,\Delta t)-CO_2^{real}(c,t+\Delta t))^2}{\sum _{c,t}(CO_2^{real}(c,t+\Delta t)-\langle CO_2^{real}(c,t+\Delta t) \rangle )^2}, \end{aligned}$$

where *t* ranges over 2001-2006 (i.e., the testing set) and $\Delta t$ is included between 1 and 15 years. $CO_2^{pred}(c,t+\Delta t,\Delta t)$ is the $\hbox {CO}_2$ prediction for country *c* at the year $t+\Delta t$ by the $\Delta t$-specific model at hand (i.e., the multiplicative regression or the RFR), while $CO_2^{real}(c,t+\Delta t)$ are the actual $\hbox {CO}_2$ emissions of country *c* at the year $t+\Delta t$. $\langle CO_2^{real}(c,t+\Delta t) \rangle$ stands as the average emissions among countries at time $t+\Delta t$, computed on the real $\hbox {CO}_2$ emissions values. The better the prediction, the closer to 1 the $R^2$ value is. This value is not country-specific and provides an overview of the quality of the prediction for a given model.

The Absolute Percentage Error for country *c* at year $t+\Delta t$ due to $\Delta t$-specific model, $APE(c,t+\Delta t,\Delta t)$, is

5 $$\begin{aligned} APE(c,t+\Delta t,\Delta t)=\left| \frac{CO_2^{pred}(c,t+\Delta t,\Delta t)-CO_2^{real}(c,t+\Delta t)}{CO_2^{real}(c,t+\Delta t)} \right| \cdot 100. \end{aligned}$$

The smaller the $APE(c,t+\Delta t,\Delta t)$ value is, the more accurate the prediction is. The Absolute Percentage Error reports information specific to the country at hand.

## Supplementary Information

Supplementary Information 1.

## Data Availability

Data sources are reported in Table 1 and Data and methods section. Data are available upon request from the corresponding author.
