# Author Correction: Forecasting national CO_2_ emissions worldwide

**DOI:** 10.1038/s41598-024-75439-5

**Published:** 2024-10-09

**Authors:** Lorenzo Costantini, Francesco Laio, Manuel Sebastian Mariani, Luca Ridolfi, Carla Sciarra

**Affiliations:** 1 CENTAI, Turin, Italy; 2 DIATI, Politecnico Di Torino, 10129 Turin, Italy; 3 URPP Social Networks, University of Zurich, CH-8050 Zurich, Switzerland; 4 Institute of Fundamental and Frontier Sciences, University of Electronic Science and Technology of China, Chengdu, 611731 People’s Republic of China

Correction to: *Scientific Reports* 10.1038/s41598-024-73060-0, published online 28 September 2024

The Acknowledgments section in the original version of this Article was incomplete.

“We thank Penny Mealy and Alexander Teytelboym for sharing with us the green products dataset."

now reads:

“We thank Penny Mealy and Alexander Teytelboym for sharing with us the green products dataset. MSM acknowledges financial support from the URPP Social Networks of the University of Zurich."

The original Article has been corrected.

