# Supplementary material for: Forecasting national CO2 emissions worldwide
Source: Sci Rep. 2024 Sep 28;14:22438. doi: 10.1038/s41598-024-73060-0 (PMC11439049; doi:10.1038/s41598-024-73060-0)
Supplement: Supplementary file 1 — Supplementary Information 1. [file 41598_2024_73060_MOESM1_ESM.pdf]

Forecasting national CO_2 emissions worldwide. Supplementary Information

Lorenzo Costantini[1,2,*], Francesco Laio[2], Manuel Sebastian Mariani[3,4], Luca Ridolfi[2], and Carla Sciarra[2]

[1]CENTAI, Turin, Italy
[2]DIATI, Politecnico di Torino, Turin, 10129, Italy
[3]URPP Social Networks, University of Zurich, Zurich, CH-8050, Switzerland
[4]Institute of Fundamental and Frontier Sciences, University of Electronic Science and Technology of China, Chengdu, 611731, People's Republic of China
[*]lorenzo.costantini@centai.eu

ABSTRACT

This document reports supplementary figures and tables to the main text, detailing the countries we considered in the analyses. Moreover, we report the key outcomes for the analyses regarding carbon dioxide emissions from a consumption perspective. Finally, we present the computation of the predictions' confidence intervals.

Here, the reader can find supplementary figures and tables cited throughout the main text.

- Table S1 reports the statistical description of the considered features.
- Table S2 provides correspondence among country ISO code, country name, income class, and regions, also detailing the type of data availability on CO_2 emissions (territorial vs. consumption).
- Figure S1 helps identify the differences between the training and testing sets. It also shows the time ranges of the predictions, exemplified for the Δt equal to 5 (yellow) and 15 (purple) years. The same scheme holds for all Δt between 1 and 15 years. Please, note that the training and testing sets do not overlap for each Δt-specific model forecasting CO_2 emissions.
- Figure S2 presents the average correlation coefficient between countries' CO_2 emissions at different time lags.
- Figure S3 shows the relative variation in CO_2 emissions.
- Table S3 reports the coefficients' values for the multiplicative regression for territorial per-capita CO_2 emissions when $\Delta t = 15$ years.
- Figure S4 shows the SHapley Additive exPlanations (SHAP)[1] for the RFR model when $\Delta t = 15$ years in case of territorial CO_2 emissions. In brief, SHAP values are additive terms to the average model forecasting that allow one to get the final output of the model explained in terms of the contribution of each variable. In Figure S4, the reader can see that high values of territorial CO_2 emissions lead to forecasting above-average predicted values; conversely, high Generalized Economic Complexity index and fitness values relate with negative contribution.
- Figure S5 shows the CO_2 projection for the multiplicative regression model from 2020 to 2035.
- Figure S6 details the RFR's predictions for China for all the models associated with any Δt. Specifically, Figure S6 shows the SHAP values for all the features, highlighting the most relevant ones.
- Figure S7 shows the Mean Absolute Percentage Error (MAPE) for the developed models on the testing set, considering territorial CO_2 emissions. We recall that MAPE is:

$$MAPE(\Delta t) = \frac{1}{6N} \sum_{c,t} \left| \frac{CO_2^{pred}(c,t+\Delta t,\Delta t) - CO_2^{real}(c,t+\Delta t)}{CO_2^{real}(c,t+\Delta t)} \right| \cdot 100, \tag{S1}$$

Table S1. Statistical description of the considered features.

Feature	Mean	Std	Minimum	Maximum
CO_2 (tons per-capita)	4.89	5.62	0.02	34.13
GDPpc (2017 constant $ at PPP)	18972	18587	465.83	100957
EnConspc (kWh per-capita)	25939	30905	267.14	199207
RenEnCons (%)	33.36	29.10	0.00	98.34
UP (%)	58.71	21.93	10.88	100.00
HDI	0.70	0.16	0.24	0.96
ECI	0.14	1.04	-2.52	2.57
F	1.44	2.36	$1.59 \cdot 10^{-4}$	77.88
GEN	15.40	17.48	0.07	144.86
GE	0.05	0.04	$1.85 \cdot 10^{-4}$	0.31
GCI	17.76	19.28	0	139.54
RDE	0.05	0.04	$5.20 \cdot 10^{-3}$	0.17

where N is the total number of countries in our dataset, $CO_2^{pred}(c, t+\Delta t)$ is the predicted CO_2 emissions for country c at year $t+\Delta t$ by the Δt-specific model (we remind that t ranges from 2001 to 2006). $CO_2^{real}(c, t+\Delta t)$ are the actual CO_2 emissions of country c at the year $t+\Delta t$. Please, note that N is multiplied by 6 because we consider 6 years (i.e., from 2001 to 2006, included) in the testing set.

- Table S4 compares the countries, methods, features, and predicted years we considered with those of previous research works.
- Figures S8-S11 and Table S5 report the same analyses shown throughout the main text on the consumption CO_2 emissions. Please note that, due to data availability, this analysis is limited to 101 countries: 4 low-, 31 lower-middle, 26 upper-middle, and 40 high-income-countries (please refer to Table S2). Moreover, the years from 2001 to 2005 (included) compose the testing set.
- Figure S12 presents the Pearson's correlation coefficient (ρ) and Variance Inflation Factor (VIF) values ($VIF = 1/(1-\rho^2)$) among all explanatory variables in the dataset. Both quantities are computed among the variables considering both the territorial and consumption CO_2 emission per person.

Table S2. Correspondence table among countries' ISO code, country name, and income class (defined according to the World Bank) and world region. Please note that: HI, LI, LMI, and UMI stand for high income, low income, lower middle income, and upper middle income, respectively. Moreover, the last two columns indicate with an "x" whether the country at hand is included in a given analysis depending on the data availability for the type of CO_2 budgeting (i.e., territorial or consumption).

ISO code	Country name	Income class	Region	Territorial CO_2 data	Consumption CO_2 data
ALB	Albania	UMI	Europe (excl. EU)	x	x
ARE	United Arab Emirates	HI	Asia (excl. China and India)	x	x
ARG	Argentina	UMI	South America	x	x
ARM	Armenia	UMI	Asia (excl. China and India)	x	x
AUS	Australia	HI	Oceania	x	x
AUT	Austria	HI	European Union	x	x
AZE	Azerbaijan	UMI	Asia (excl. China and India)	x	x
BEL	Belgium	HI	European Union	x	x
BEN	Benin	LMI	Africa	x	x
BGD	Bangladesh	LMI	Asia (excl. China and India)	x	x
BGR	Bulgaria	UMI	European Union	x	x
BLR	Belarus	UMI	Europe (excl. EU)	x	x
BOL	Bolivia	LMI	South America	x	x

Continued on next page

Table S2 – continued from previous page

ISO code	Country name	Income class	Region	Territorial CO_2 data	Consumption CO_2 data
BRA	Brazil	UMI	South America	x	x
CAN	Canada	HI	North America (excl. US)	x	x
CHE	Switzerland	HI	Europe (excl. EU)	x	x
CHL	Chile	HI	South America	x	x
CHN	China	UMI	China	x	x
CIV	Côte d'Ivoire	LMI	Africa	x	x
CMR	Cameroon	LMI	Africa	x	x
COD	Congo, the Dem. Rep. of	LI	Africa	x	
COG	Congo	LMI	Africa	x	
COL	Colombia	UMI	South America	x	x
CRI	Costa Rica	UMI	North America (excl. US)	x	x
CZE	Czech Republic	HI	European Union	x	x
DEU	Germany	HI	European Union	x	x
DNK	Denmark	HI	European Union	x	x
DOM	Dominican Republic	UMI	North America (excl. US)	x	x
DZA	Algeria	LMI	Africa	x	
ECU	Ecuador	UMI	South America	x	x
EGY	Egypt	LMI	Africa	x	x
ESP	Spain	HI	European Union	x	x
EST	Estonia	HI	European Union	x	x
FIN	Finland	HI	European Union	x	x
FRA	France	HI	European Union	x	x
GAB	Gabon	UMI	Africa	x	
GBR	United Kingdom	HI	Europe (excl. EU)	x	x
GHA	Ghana	LMI	Africa	x	x
GIN	Guinea	LMI	Africa	x	x
GRC	Greece	HI	European Union	x	x
GTM	Guatemala	UMI	North America (excl. US)	x	x
HKG	Hong Kong SAR, China	HI	Asia (excl. China and India)	x	x
HND	Honduras	LMI	North America (excl. US)	x	x
HRV	Croatia	HI	European Union	x	x
HTI	Haiti	LMI	North America (excl. US)	x	
HUN	Hungary	HI	European Union	x	x
IDN	Indonesia	UMI	Asia (excl. China and India)	x	x
IND	India	LMI	India	x	x
IRL	Ireland	HI	European Union	x	x
IRN	Iran	LMI	Asia (excl. China and India)	x	x
ISR	Israel	HI	Asia (excl. China and India)	x	x
ITA	Italy	HI	European Union	x	x
JAM	Jamaica	UMI	North America (excl. US)	x	x
JOR	Jordan	LMI	Asia (excl. China and India)	x	x
JPN	Japan	HI	Asia (excl. China and India)	x	x
KAZ	Kazakhstan	UMI	Asia (excl. China and India)	x	x
KEN	Kenya	LMI	Africa	x	x
KGZ	Kyrgyzstan	LMI	Asia (excl. China and India)	x	x
KHM	Cambodia	LMI	Asia (excl. China and India)	x	x
KOR	South Korea	HI	Asia (excl. China and India)	x	x
KWT	Kuwait	HI	Asia (excl. China and India)	x	x
LAO	Lao PDR	LMI	Asia (excl. China and India)	x	x
LKA	Sri Lanka	LMI	Asia (excl. China and India)	x	x
LTU	Lithuania	HI	European Union	x	x

Continued on next page

Table S2 – continued from previous page

ISO code	Country name	Income class	Region	Territorial CO_2 data	Consumption CO_2 data
LVA	Latvia	HI	European Union	x	x
MAR	Morocco	LMI	Africa	x	x
MDA	Moldova	UMI	Europe (excl. EU)	x	
MEX	Mexico	UMI	North America (excl. US)	x	x
MKD	North Macedonia	UMI	Europe (excl. EU)	x	
MLI	Mali	LI	Africa	x	
MMR	Myanmar	LMI	Asia (excl. China and India)	x	
MNG	Mongolia	LMI	Asia (excl. China and India)	x	x
MOZ	Mozambique	LI	Africa	x	x
MRT	Mauritania	LMI	Africa	x	
MUS	Mauritius	UMI	Africa	x	x
MWI	Malawi	LI	Africa	x	x
MYS	Malaysia	UMI	Asia (excl. China and India)	x	x
NER	Niger	LI	Africa	x	
NIC	Nicaragua	LMI	North America (excl. US)	x	x
NLD	Netherlands	HI	European Union	x	x
NOR	Norway	HI	Europe (excl. EU)	x	
NPL	Nepal	LMI	Asia (excl. China and India)	x	x
NZL	New Zealand	HI	Oceania	x	x
PAK	Pakistan	LMI	Asia (excl. China and India)	x	x
PAN	Panama	HI	North America (excl. US)	x	
PER	Peru	UMI	South America	x	x
PHL	Philippines	LMI	Asia (excl. China and India)	x	x
PNG	Papua New Guinea	LMI	Oceania	x	
POL	Poland	HI	European Union	x	x
PRT	Portugal	HI	European Union	x	x
PRY	Paraguay	UMI	South America	x	x
ROU	Romania	HI	European Union	x	x
RUS	Russian Federation	UMI	Asia (excl. China and India)	x	x
SAU	Saudi Arabia	HI	Asia (excl. China and India)	x	x
SDN	Sudan	LI	Africa	x	
SEN	Senegal	LMI	Africa	x	x
SGP	Singapore	HI	Asia (excl. China and India)	x	x
SLE	Sierra Leone	LI	Africa	x	
SLV	El Salvador	UMI	North America (excl. US)	x	x
SVK	Slovak Republic	HI	European Union	x	x
SVN	Slovenia	HI	European Union	x	x
SWE	Sweden	HI	European Union	x	x
TGO	Togo	LI	Africa	x	x
THA	Thailand	UMI	Asia (excl. China and India)	x	x
TJK	Tajikistan	LMI	Asia (excl. China and India)	x	x
TTO	Trinidad and Tobago	HI	South America	x	x
TUN	Tunisia	LMI	Africa	x	x
TUR	Turkey	UMI	Asia (excl. China and India)	x	x
TZA	Tanzania	LMI	Africa	x	x
UGA	Uganda	LI	Africa	x	x
UKR	Ukraine	LMI	Europe (excl. EU)	x	x
URY	Uruguay	HI	South America	x	x
USA	United States	HI	US	x	x
VNM	Vietnam	LMI	Asia (excl. China and India)	x	x
ZAF	South Africa	UMI	Africa	x	x

Continued on next page

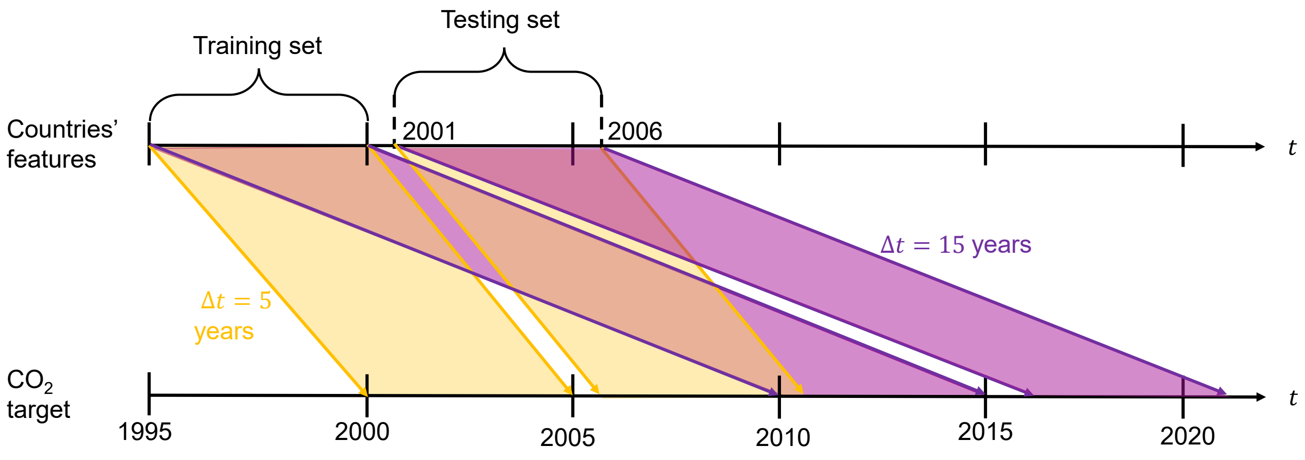

Figure S1. A qualitative scheme showing the training and testing sets for two Δt-specific models (yellow refers to $\Delta t = 5$ years and purple to $\Delta t = 15$ years). Specifically, for each Δt, the model receives as input the countries' features presented in Table 1 of the main text for all countries and years in the training set to fit the CO_2 emissions at time $t + \Delta t$. The same procedure holds for the models' validation in the testing set. For each Δt ranging from 1 to 15 years, the training and testing sets do not overlap. Please, note that the target CO_2 emissions for 2021 are retrieved from the Global Carbon Budget[2].

Table S2 – continued from previous page

ISO code	Country name	Income class	Region	Territorial CO_2 data	Consumption CO_2 data
ZMB	Zambia	LMI	Africa	x	x
ZWE	Zimbabwe	LMI	Africa	x	x

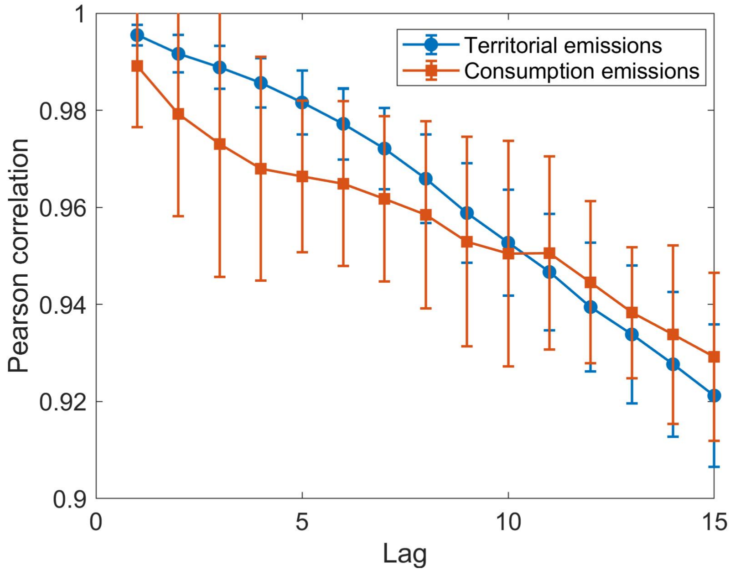

Figure S2. Auto-correlation of per-capita territorial (blue circles) and consumption (red squares) CO_2 emissions as a function of the time lag. Each marker reports the country-averaged correlation values and vertical whiskers show ± 1 standard deviation.

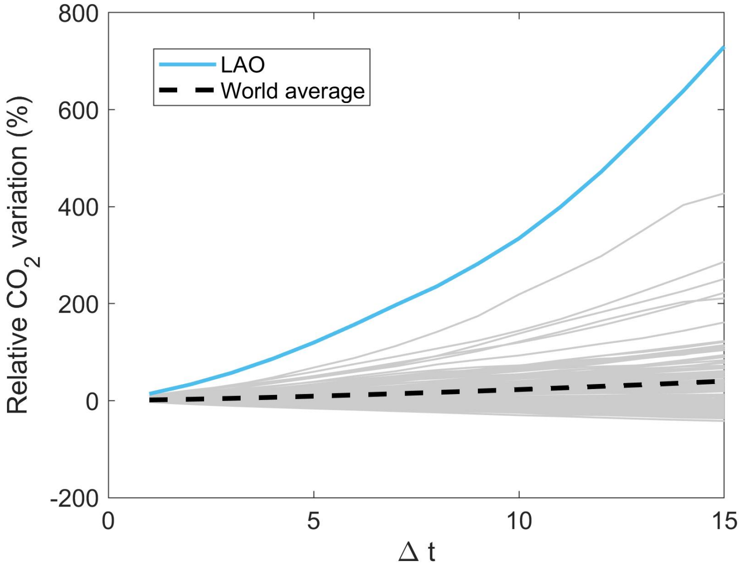

Figure S3. Average relative CO_2 variation in percentage as function of Δt (i.e., $100 \cdot (CO_2^{real}(c,t+\Delta t) - CO_2^{real}(c,t))/CO_2^{real}(c,t))$. The thin grey lines show the CO_2 changes for all countries in the dataset, the thick light blue line refers to Laos (LAO), and the black dashed thick line reports the world average. Please, note that the average variation is computed onto all the possible couples of $CO_2^{real}(c,t+\Delta t)$ and $CO_2^{real}(c,t)$ within our territorial emissions data, at fixed Δt.

Table S3. Coefficients of the multiplicative regressive model for $\Delta t = 15$ years, considering the territorial CO_2 emissions per capita. Features are sorted according to a forward step-wise feature selection algorithm from the most (ranking = 1) to the least important (ranking = 12) for this specific model. Please, refer to Table 1 of the main text for the variables' description. The asterisks indicate p-value ranges (*: p-value < 10%, **: p-value < 5%, and ***: p-value < 1%).

Ranking	Multiplicative regression	Coefficient
1	CO_2	0.83***
2	ECI	-0.38***
3	RenEnCons	-0.063***
4	F	0.022
5	HDI	0.27*
6	GDPpc	-0.063*
7	RDE	-0.085**
8	GEN	0.087
9	GCI	-0.096**
10	EnConspc	0.033
11	GE	0.017
12	UP	-0.009

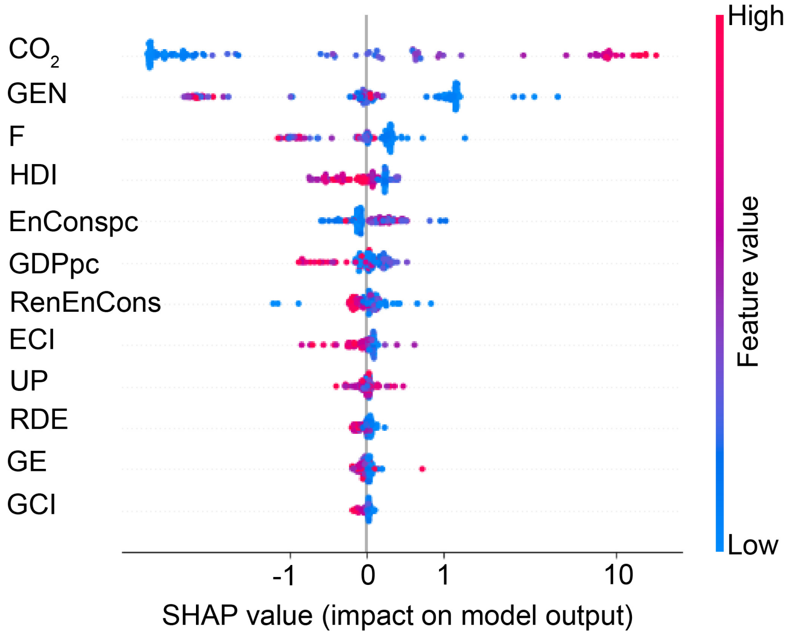

Figure S4. Description of how the considered explanatory variables affect the predictions of the Random Forest Regressor (RFR) when $\Delta t = 15$ years for territorial CO_2 emissions per-capita using the SHapley Additive exPlanations (SHAP)[1]. The variables are sorted in descending order, from the most important (the CO_2) to the least important (the Green Complexity Index, GCI[3]). For each sample in the training set, the figure shows the impact on the prediction of each variable, called the SHAP value. We recall that the final RFR output for a country is increased (reduced) by positive (negative) SHAP values compared with the average prediction of the RFR among all countries[1]. The color of each dot refers to the value of the feature.

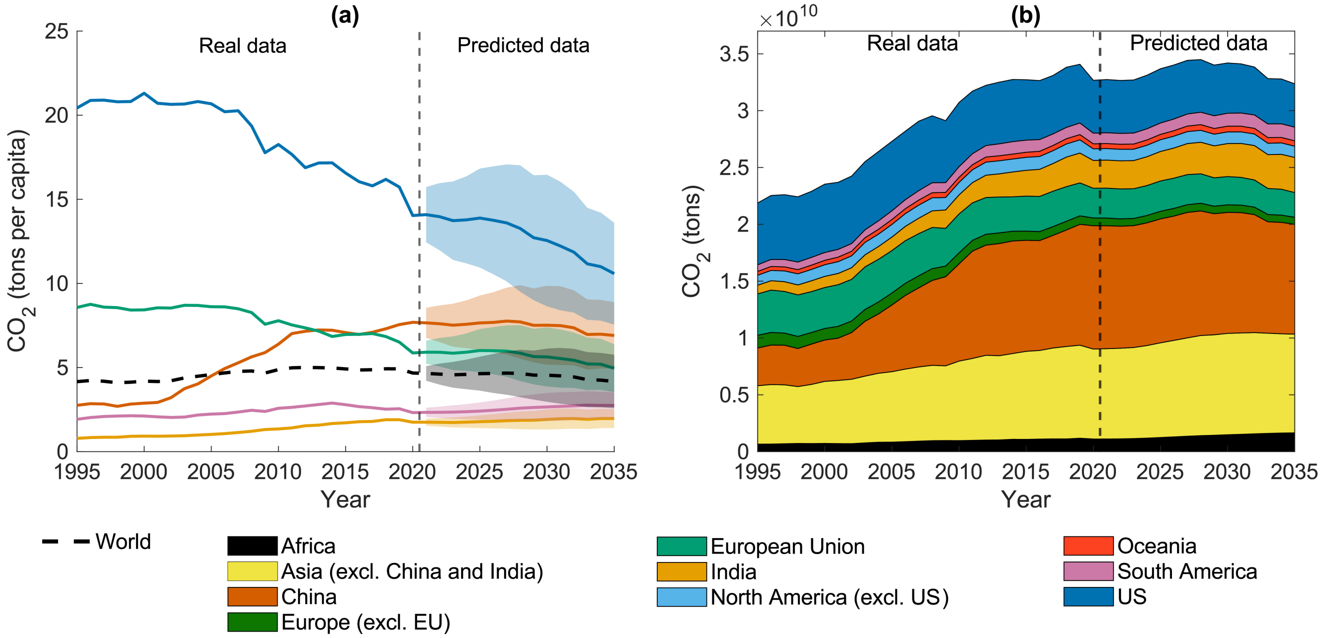

Figure S5. CO_2 projections until 2035 by the Δt-specific models through a multiplicative regression modeling approach. Panel (a) shows the per-capita CO_2 projection for some specific countries and regions: China, the European Union, India, South America, and the United States (US), and the world average (black thick dashed line). Panel (b) reports the CO_2 emissions at country or region scale for countries aggregated in regions as reported in Table S2. Please, note that panel (b) considers the central value of the forecasting. In both panels, the dashed thin vertical line separates the actual data from the predicted ones.

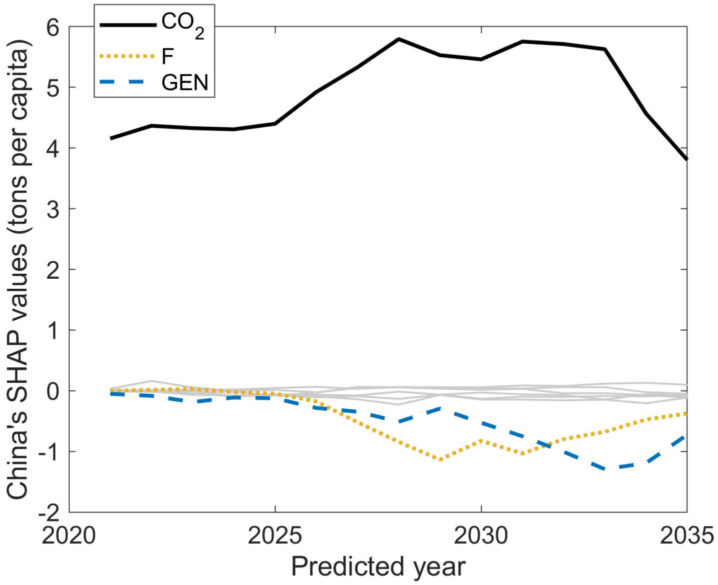

Figure S6. RFR's SHAP values for China's prediction. Grey thin lines show the SHAP values for all the features, and we highlighted those presenting the high impacts on model output: CO_2 (black solid line), fitness (dotted yellow line), and Generalized Economic Complexity index (GEN, blue dashed line). Here, we considered the RFR random state equal to 42 and 200 trees.

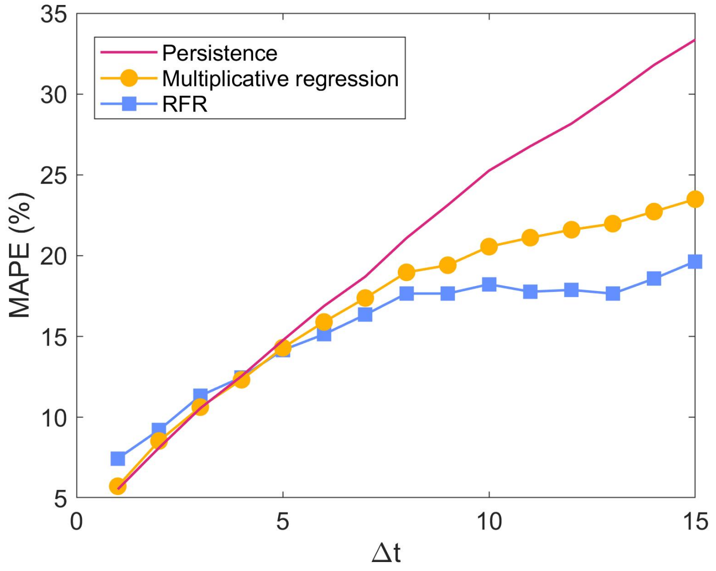

Figure S7. Mean Absolute Percentage Error (Equation (S1)) as a function of the Δt (i.e., the developed models) computed on territorial CO_2 emissions. To produce these results, for the RFR, use a random state equal to 42 and 200 trees.

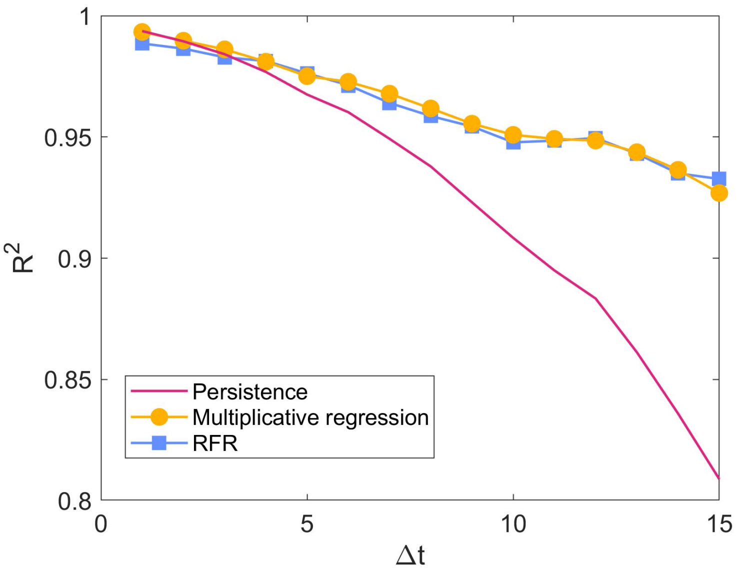

Figure S8. The determination coefficient (R^2, Equation (4) of the main text) computed between real and predicted CO_2 emissions in the testing set (i.e., input data between 2001 and 2005) for each Δt-specific model. Please note that the determination coefficient is computed onto the logarithm of the consumption emissions to balancedly weigh the residues of all countries, which span over different orders of magnitude. (To produce these results, we considered a Random Forest Regressor with 200 trees and a random state equal to 42).

Table S4. Comparison among our work and previous studies providing CO_2 projection considering the number of countries, used methods, features, emissions target, and predicted years.

Reference	Number of countries	Method	Features	Target CO_2 emissions	Predicted years
Köne and Bürke (2010)[4]	25 (Australia, Brazil, Canada, China, France, Germany, India, Indonesia, Islamic Republic of Iran, Italy, Japan, Kazakhstan, Mexico, Poland, Russian Federation, Saudi Arabia, South Africa, South Korea, Spain, Taiwan, Thailand, Turkey, Ukraine, United Kingdom, United States)	Country-specific trend analysis	CO_2	National CO_2 emissions.	2030 (CO_2 projections are provided for 10 countries: Australia, Brazil, India, Indonesia, Islamic Republic of Iran, Mexico, Saudi Arabia, South Africa, Turkey).
Gao et al. (2021)[5]	3 (China, Japan, United States)	Country-specific Grey-Riccati model	GDP, energy consumption, trade openness	National CO_2 emissions	2020, 2021, 2022, 2023, 2024, 2025
Karakurt and Aydin (2023)[6]	9 (Brazil, China, India, Indonesia, Mexico, Nigeria, Russian Federation, South Africa, Turkey)	Country-specific regression models	GDPpc, energy consumption, urban population, total population	National fossil fuels-related CO_2 emissions	2025, 2030, 2035, 2040, 2045
Costantini et al. (2024)	117 (see Table S2)	Global models: multiplicative regression and Random Forest Regressor	CO_2, GDPpc, energy consumption, renewable energies, urbam population, HDI, Economic Complexity, Green Economy, RDE (see Table 1 of the main text)	Per-capita CO_2 emissions. National estimations are obtained by scaling for population projections of the United Nations.	2021-2035, all years

Table S5. Mean variables' ranking for each forecasting model among all the considered Δt for consumption CO_2 emissions. Variables' order is computed according to a forward step-wise feature selection algorithm for the regressive model and the mean decrease impurity for the Random Forest Regressor (RFR).

Ranking	Multiplicative regression	RFR
1	CO_2	CO_2
2	EnConspc	EnConspc
3	ECI	RenEnCons
4	RenEnCons	HDI
5	GEN	GDPpc
6	HDI	F
7	F	GE
8	GDPpc	ECI
9	RDE	UP
10	GCI	RDE
11	UP	GEN
12	GE	GCI

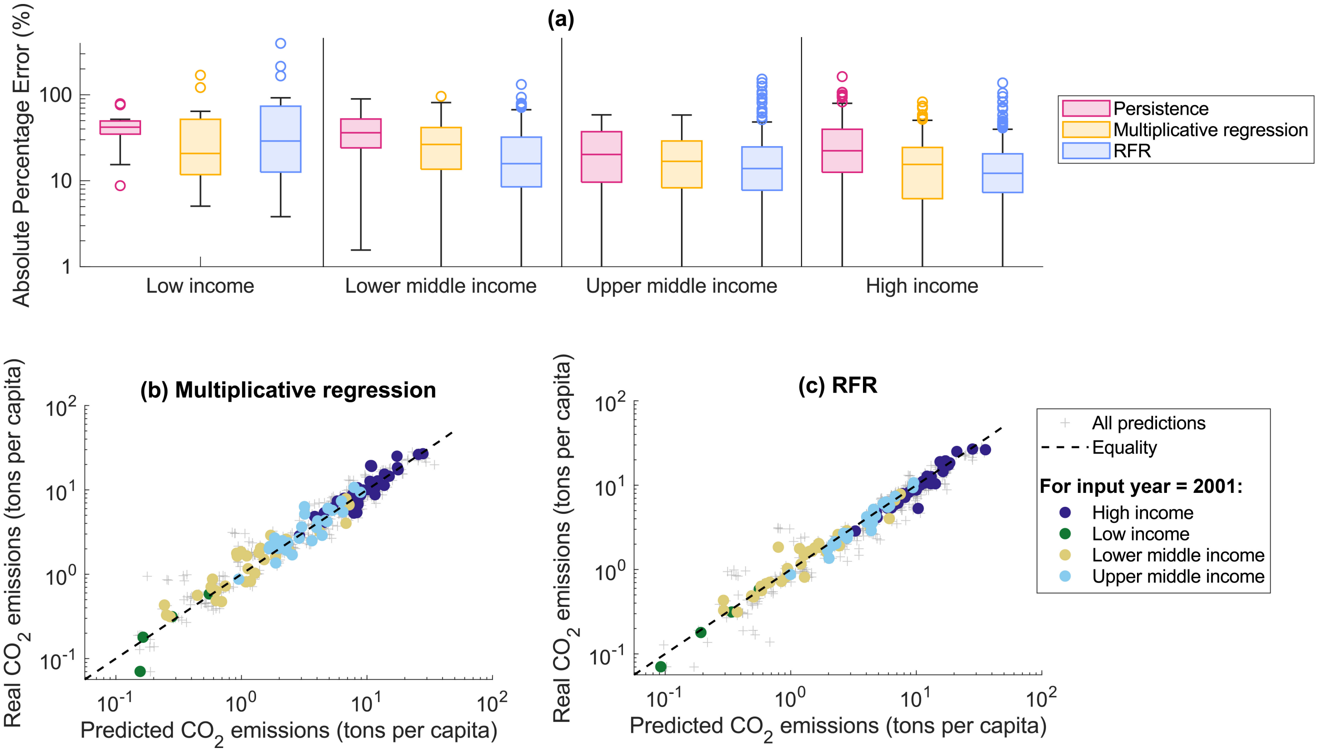

Figure S9. Accuracy performances for the models associated with $\Delta t = 15$ years on the testing set (i.e., 2001-2005) for consumption CO_2 emissions. Panel (a) shows the Absolute Percentage Errors (Equation (5)) for all countries (grouped by income class according to the World Bank classification) and samples in the testing set. The vertical black lines help the reader to separate the boxes according to the different income classes. For a given income class, each model-specific box shows the median, the lower and upper quartiles (box), any outliers (computed using the interquartile range, void circles), and the minimum and maximum values that are not outliers (whiskers) and are color-coded according to its reference model. To improve the readability of the boxes, the y-axis was cut at 1%. Panels (b) and (c) compare actual and predicted CO_2 emissions for the multiplicative regression and RFR models, respectively. Grey pluses (+) represent all model outputs for $\Delta t = 15$ years for input features in the testing set (i.e., 2001-2005). Filled dots highlight models' predictions for input year equal to 2001 (i.e., 2016 forecasting). The color of the dots describes the income class the countries belong to. The dashed lines show the perfect match between actual and predicted values. (RFR results refer to a random state equal to 42 and 200 trees.)

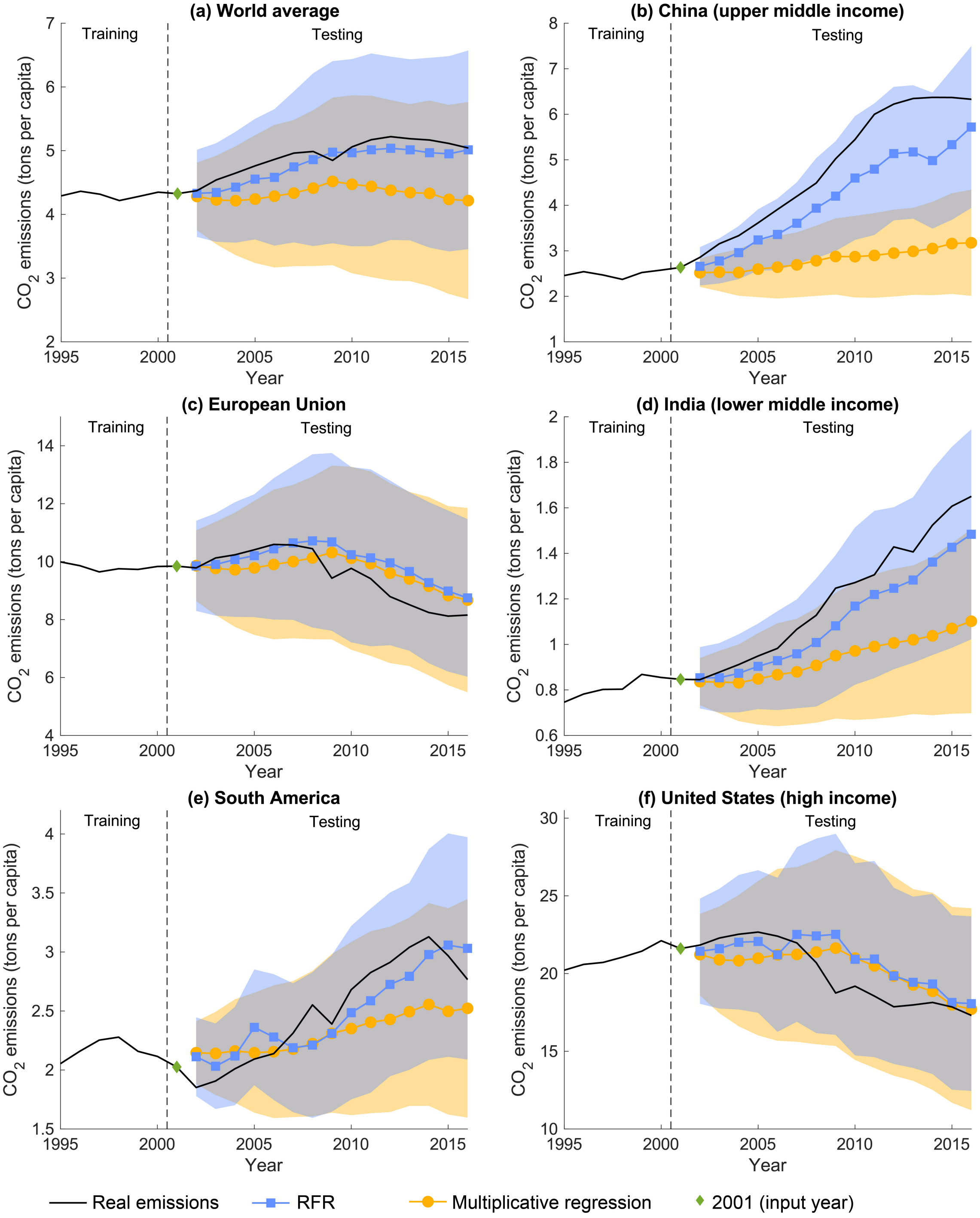

Figure S10. Comparison among countries' and regions' actual consumption CO_2 emissions and the predicted ones by the Random Forest Regressor –RFR– model (blue squares) and multiplicative regression approach (yellow circles) for world average (panel (a)), China (panel (b)), European Union (panel (c)), India (panel (d)), South America (panel (e)), and United States (panel (f)). Shaded regions, color-coded according to the model they refer to, show confidence intervals associated with each prediction at 80% (overlapping confidence intervals between the models are in grey), and the green diamond highlights the CO_2 emissions in 2001 for the country at hand (i.e., the CO_2 input data to the models). The dashed vertical lines separate the training from the testing period. (The RFR predictions are obtained considering a random state equal to 42 and 200 trees.)

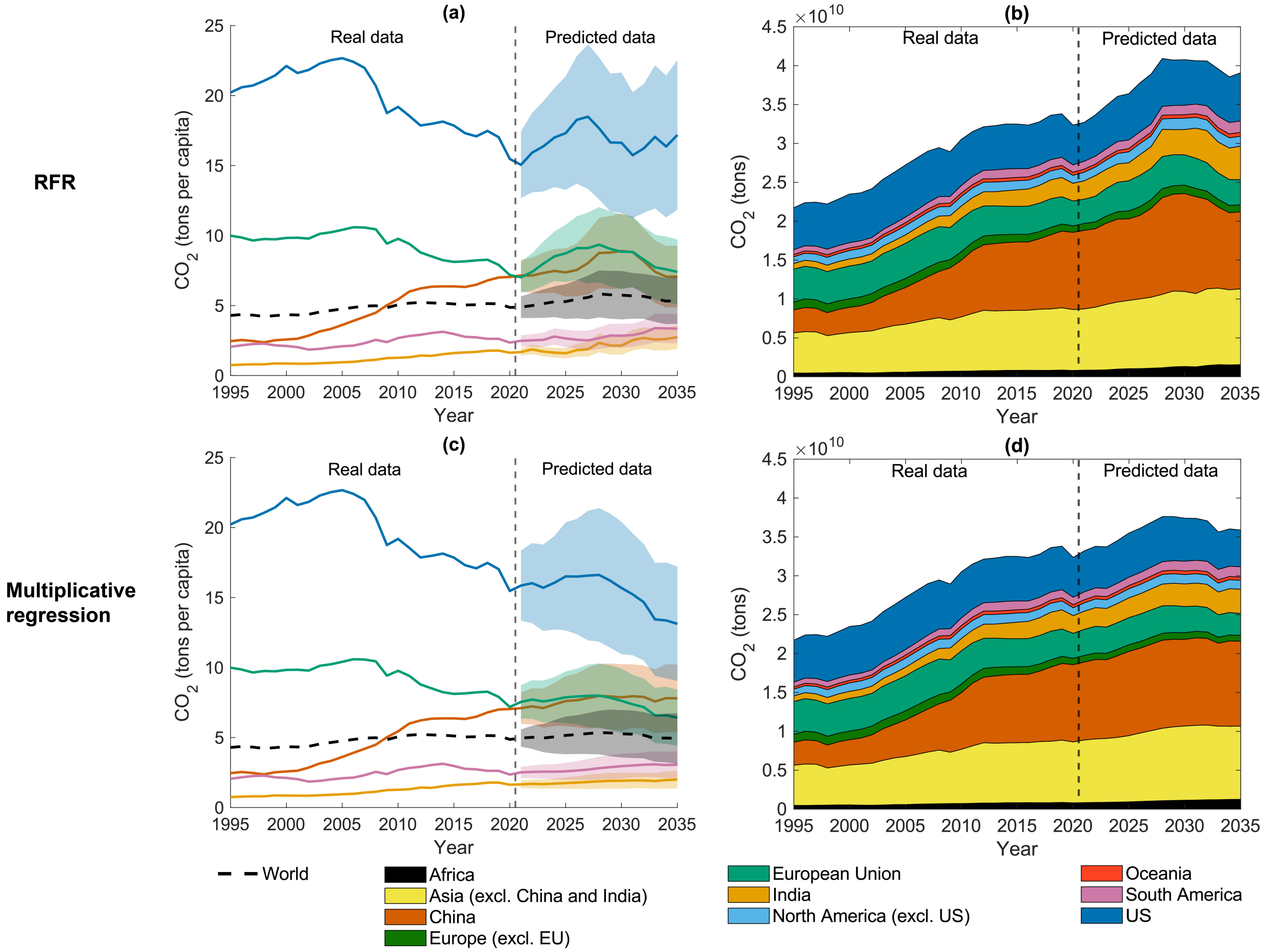

Figure S11. Consumption CO_2 projections from 2020 to 2035 by the Random Forest Regressor (panels (a) and (b)) and multiplicative regression (panels (c) and (d)). Panels (a) and (c) show the per-capita CO_2 projection of the world average (black thick dashed line) for some specific countries and regions: China, the European Union, India, South America, and the United States (US). Panels (b) and (d) report the CO_2 emissions at country or region scale for countries aggregated in regions as reported in Table S2. Please, note that panels (b) and (d) consider the central value of the forecasting. In each panel, the dashed thin vertical line separates the actual data from the predicted ones. (To produce these results, for the RFR, use a random state equal to 42 and 200 trees.)

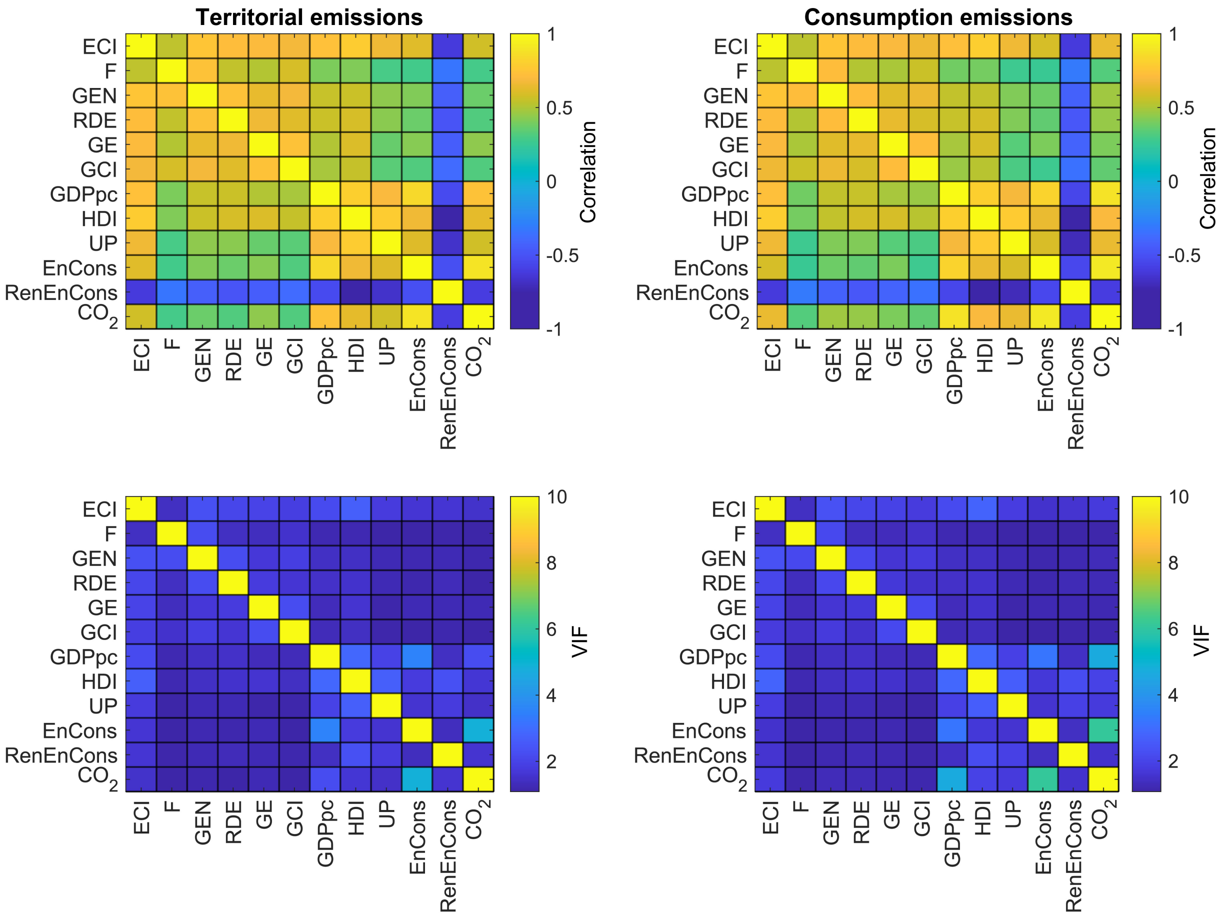

Figure S12. Correlation (top panels) and VIF (bottom panels) values across the variables in the dataset for territorial (left panels) and consumption (right panels) CO_2 emissions. For VIF values (bottom panels), the infinite values along the principal diagonal have been set to 10 to improve readibility.

Confidence interval

Confidence intervals associated with CO_2 predictions were computed supposing a normal distribution around the predicted CO_2 value (CO_2^{pred}), described by the following equation

$$P(x,\mu,\sigma) = \frac{1}{\sqrt{2\pi\sigma^2}} e^{-\frac{(x-\mu)^2}{2\sigma^2}}, \tag{S2}$$

where x are the CO_2 emission values. The parameter μ is the central value of the distribution and it is assumed equal to the predicted CO_2 value for country c by a model for a specific Δt (i.e., $\mu = CO_2^{pred}(c, t+\Delta t, \Delta t)$).

The standard deviation (σ) was computed as follows. Firstly, for each country c and Δt-specific model, we calculated the average root mean square error ($s(c,\Delta t)$ as

$$s(c,\Delta t) = \sqrt{\frac{1}{6} \cdot \sum_{t=2001}^{2006} (CO_2^{pred}(c, t+\Delta t, \Delta t) - CO_2^{real}(c, t+\Delta t))^2}, \tag{S3}$$

where $CO_2^{real}(c, t+\Delta t)$ is the actual CO_2 emissions of country c at year $t+\Delta t$. Secondly, we introduced a country- and model-specific coefficient of variation ($\widetilde{CV}(c,\Delta t)$), defined as

$$\widetilde{CV}(c,\Delta t) = \frac{s(c,\Delta t)}{(1/6) \cdot \sum_{t=2001}^{2006} CO_2^{pred}(c, t+\Delta t, \Delta t)}. \tag{S4}$$

Please, note that the denominator of Equation (S4) is the mean CO_2 emissions predicted by the Δt-specific model for country c in the testing set. Thirdly, we averaged $\widetilde{CV}(c,\Delta t)$ among all countries, obtaining a model-specific coefficient of variation ($CV(\Delta t)$). In mathematical terms:

$$CV(\Delta t) = \frac{1}{N} \cdot \sum_c \widetilde{CV}(c,\Delta t), \tag{S5}$$

where N is the number of countries considered in the analyses (i.e., 117 for territorial CO_2 emissions). Finally, the standard deviation of country c for the CO_2 prediction at time $t+\Delta t$ corresponding to the Δt-specific model ($\sigma(c, t+\Delta t, \Delta t)$) is:

$$\sigma(c, t+\Delta t, \Delta t) = CV(\Delta t) \cdot CO_2^{pred}(c, t+\Delta t, \Delta t). \tag{S6}$$

To verify that our assumption of normal distribution (Equation (S2)) can provide a reliable estimate of the predictions' confidence interval, we computed the probability of not overcoming the actual value (q) for each country as:

$$q(c, t+\Delta t, \Delta t) = \int_0^{CO_2^{real}(c,t+\Delta t)} P(x, CO_2^{pred}(c, t+\Delta t, \Delta t), \sigma(c, t+\Delta t, \Delta t))dx, \tag{S7}$$

Figure S13 compares the actual (obtained using Equation (S7)) and empirical q, considering all countries and models for all years in the testing set. The uniformity plots for the multiplicative regression (panel S13a) and RFR (panel S13b) models show that the empirical and actual probability of not overcoming the actual CO_2 emissions are coherent: the lines referring to the predictive models are generally within the Kolmogorov bands at 5%. Some years slightly fall outside the Kolmogorov bands, but these are few and isolated cases. Therefore, we used the mathematical framework described above to compute the confidence interval at 80% shown in Figures 3 and 4 of the main text.

References

1. Lundberg, S. M. & Lee, S.-I. A unified approach to interpreting model predictions. *Adv. Neural Inf. Process. Syst.* **30** (2017).
2. Friedlingstein, P. *et al.* Global carbon budget 2021. *Earth Syst. Sci. Data* **14**, 1917–2005 (2022).
3. Mealy, P. & Teytelboym, A. Economic complexity and the green economy. *Res. Policy* **51**, 103948 (2022).
4. Köne, A. Ç. & Büke, T. Forecasting of CO_2 emissions from fuel combustion using trend analysis. *Renew. Sustain. Energy Rev.* **14**, 2906–2915 (2010).
5. Gao, M., Yang, H., Xiao, Q. & Goh, M. A novel fractional grey Riccati model for carbon emission prediction. *J. Clean. Prod.* **282**, 124471 (2021).
6. Karakurt, I. & Aydin, G. Development of regression models to forecast the CO_2 emissions from fossil fuels in the BRICS and MINT countries. *Energy* **263**, 125650 (2023).

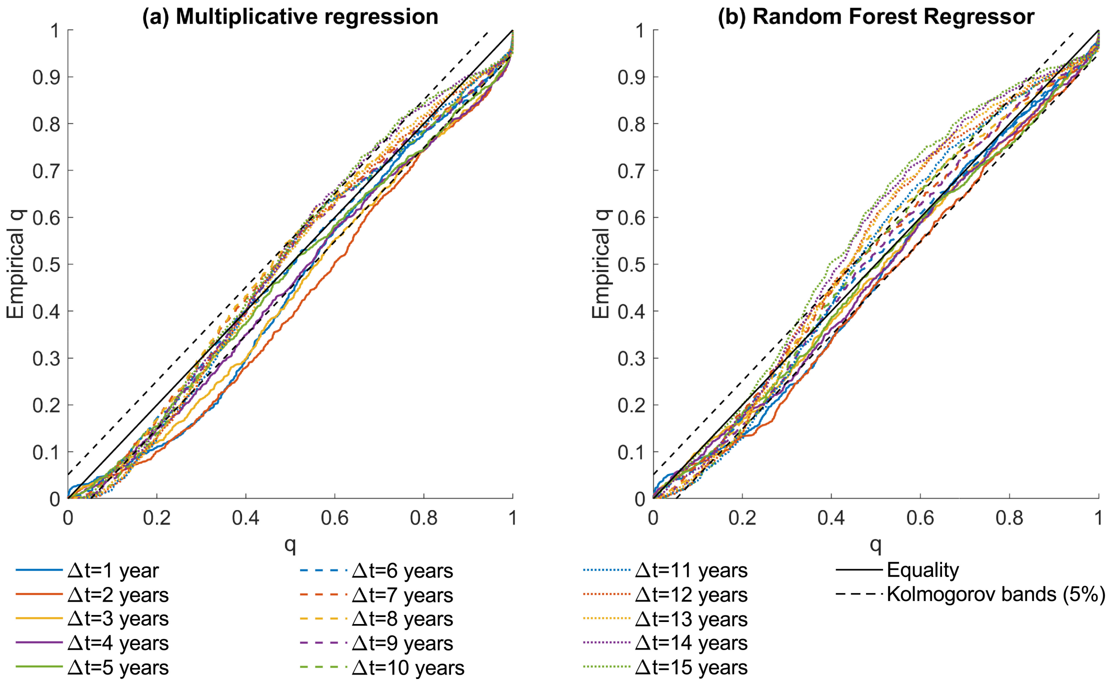

Figure S13. Uniformity plots between the probability of not overcoming the actual CO_2 emissions (q, x-axis, Equation (S7)) and the empirical q (y-axis). Panel (a) shows the results for the multiplicative regression and panel (b) reports the results for the Random Forest Regressor model. All years in the testing set are considered in these uniformity plots. In both panels, each colored line refers to the results of a specific model identified by the Δt. The black solid line reports the equality line, while the black dashed lines display the Kolmogorov bands at 5%. (For reproducibility, the RFR model has 200 trees and a random state equal to 42.)

Acknowledgements

We thank Penny Mealy and Alexander Teytelboym for sharing with us the green products dataset.

Author contributions statement

L.C., F.L, M.S.M, L.R., and C.S. conceived and designed the study. L.C. performed the experiments. L.C., F.L, M.S.M., L.R., and C.S. analysed the results. L.C. wrote the manuscript and made all the figures. F.L, M.S.M., L.R., and C.S. edited the manuscript. All authors reviewed the manuscript.

Additional information

The authors have no competing interests to disclose.
